# DyProL: Dynamic Ensemble Representation Learning for Protein–Nucleic Acid Binding Site Prediction

**DOI:** 10.1002/advs.77501

**Published:** 2026-09-03

**Authors:** Pengpai Li, Yiman Liu, Liya Liang, Rongming Liu

**Affiliations:** ^1^ MOE Key Laboratory of Bio‐Intelligent Manufacturing School of Bioengineering Dalian University of Technology Dalian Liaoning China; ^2^ Ningbo Institute of Dalian University of Technology Ningbo Zhejiang China

**Keywords:** binding site prediction, conformational ensembles, dynamic protein representation, geometric deep learning

## Abstract

Protein–nucleic acid interactions play central roles in gene regulation and cellular function, and extensive efforts have been devoted to predicting nucleic acid binding sites from protein structures. However, protein–nucleic acid recognition is inherently dynamic, whereas most existing computational approaches rely on single static conformations, limiting their ability to capture conformational heterogeneity underlying binding. Here, we present DyProL, an ensemble‐based conformational representation learning framework that models proteins as ensembles of conformations sampled from equilibrium‐like structural distributions. DyProL learns dynamic structural features through iterative aggregation of intra‐ and inter‐conformation geometric information, enabling representation of both local structural context and global conformational variability. Across multiple benchmarks, DyProL consistently outperforms state‐of‐the‐art methods in nucleic acid binding site prediction, with particularly pronounced improvements under realistic settings using predicted or apo‐like structures, where static methods degrade substantially. These results establish dynamic ensemble‐based representations as a general and scalable paradigm for structure‐based protein modeling, providing a foundation for improving a broad range of protein function prediction tasks.

## Graphical abstract

Protein function is represented as a dynamic conformational ensemble rather than a single static structure. A multi‐conformation geometric attention framework aligns, clusters, and learns representative states to capture residue‐ and ensemble‐level signals. Integrating structural dynamics improves interpretable protein‐NA binding prediction and reveals how conformational diversity contributes to molecular recognition.

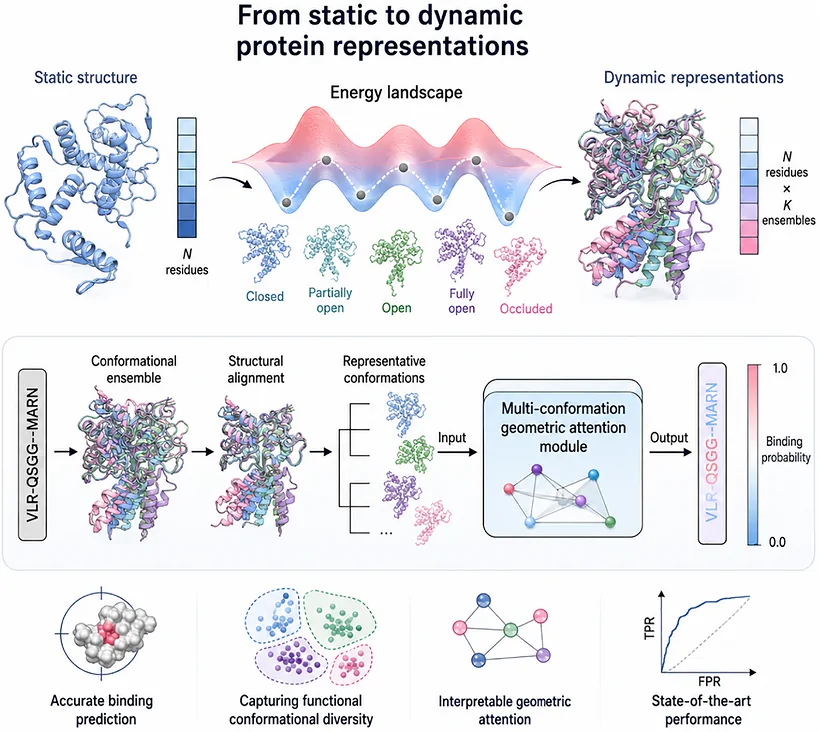

## 1 Introduction

Nucleic acid binding proteins (NBPs) are central regulators of cellular information flow, governing transcriptional programs, chromatin architecture, RNA maturation, translation, and viral genome integration [1, 2]. Therefore, accurately identifying nucleic acid binding sites is critical for mechanistic dissection of gene regulation, reliable functional annotation of uncharacterized proteins, and the rational design of genome engineering, antiviral, and synthetic biology strategies [3, 4].

Machine learning–based prediction of protein–nucleic acid binding sites critically depends on the molecular representation of proteins. Informative representations must capture structural geometry, physicochemical properties [5], and evolutionary constraints that collectively govern nucleic acid recognition, whether derived from primary sequences [6] or three‐dimensional (3D) structures [7]. Early computational frameworks predominantly relied on sequence‐derived features, leveraging evolutionary conservation and domain‐specific motifs characteristic of NBPs [8, 9, 10, 11]. The rapid expansion of experimentally resolved structures [12] and large‐scale structure prediction resources [13] has since enabled structure‐informed learning paradigms that explicitly model the spatial organization underlying nucleic acid recognition. Most contemporary structure‐based approaches represent proteins as static geometric entities, including residue‐level graphs [14, 15, 16], surface or atomic point clouds [17, 18, 19], and 3D voxelized grids [20, 21]. By leveraging geometric patterns embedded in protein structures, these models have achieved strong predictive performance in binding site identification tasks.

However, most existing approaches implicitly assume that a single static conformation sufficiently captures the structural determinants of nucleic acid recognition. This assumption overlooks a fundamental property of proteins: they are intrinsically dynamic systems, whose functional competence emerges from ensembles of interconverting conformations rather than a single structural snapshot [22, 23, 24]. Such conformational heterogeneity is especially critical in protein–nucleic acid recognition. DNA‐binding proteins often undergo induced‐fit or conformational selection processes upon ligand engagement, involving domain rearrangements, helix repositioning, and large‐scale deformation around DNA duplexes [25, 26, 27]. This type of large‐scale structural plasticity is characteristic of many transcription factors, which dynamically adapt to the DNA helix during sequence‐specific recognition. RNA‐binding proteins likewise exhibit intrinsic conformational dynamics, but these are often manifested through more localized and modular structural adjustments. Many RBPs employ pre‐organized domains, such as RRMs and KH domains, that undergo flexible loop rearrangements, side‐chain reorientations, and subtle backbone shifts to accommodate diverse RNA targets [28, 29, 30]. These local dynamics are further shaped by the structural properties of RNA, whose secondary structures often exhibit higher kinetic stability and defined folding barriers [31]. Together, these observations indicate that both DNA‐ and RNA‐binding interfaces are governed by conformational heterogeneity, albeit operating at different structural scales. This distinction suggests that dynamic ensemble modeling may capture complementary aspects of binding mechanisms across these systems, and provide a mechanistic basis for its differential impact in binding site prediction tasks [32].

Despite the well‐established role of conformational dynamics in nucleic acid recognition, explicit integration of structural heterogeneity into learning‐based binding site prediction has remained largely unexplored. A principal barrier has been the limited availability of experimentally resolved multi‐state structures, coupled with the absence of scalable computational frameworks for representing conformational variability. Recent advances in generative structural modeling have begun to alleviate this constraint. A new class of deep models can efficiently sample diverse protein conformations without recourse to explicit molecular dynamics simulations [33, 34, 35]. By enabling scalable generation of conformational ensembles, these methods provide a practical route to approximating protein structural heterogeneity. Such developments open the possibility of incorporating dynamic ensemble representations into nucleic acid binding site prediction.

**FIGURE 1:**
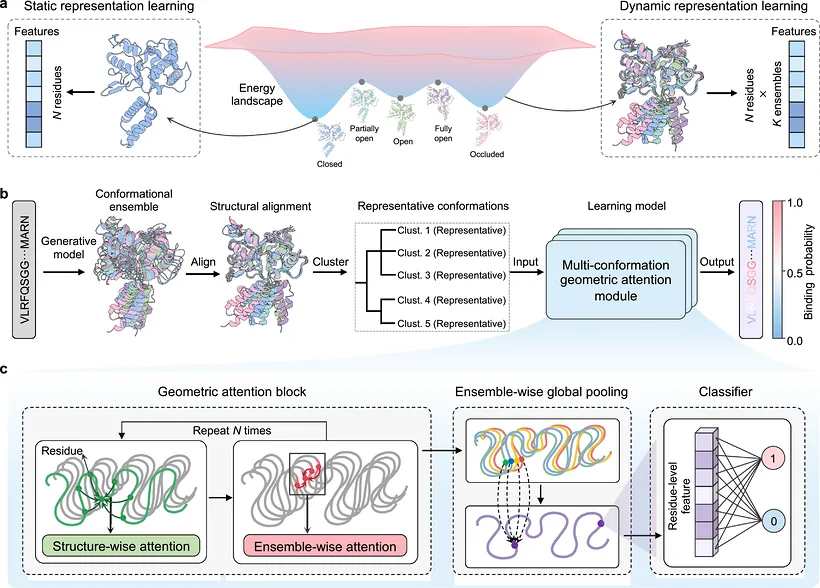
Framework of DyProL. (a) Static versus dynamic protein representation learning. Existing methods encode proteins from a single structural snapshot, overlooking the underlying conformational landscape. DyProL instead models proteins as ensembles of interconverting conformations sampled from equilibrium‐like energy distributions, enabling representation of conformational heterogeneity. (b) DyProL pipeline. For each protein, conformational ensembles are generated, structurally aligned, and clustered to obtain representative states. These states are jointly processed by a multi‐conformation learning framework to predict residue‐level binding probabilities. (c) Model architecture. DyProL employs geometric attention blocks to capture structure‐wise (intra‐conformation) and ensemble‐wise (inter‐conformation) dependencies. An ensemble‐level aggregation module integrates information across conformations, followed by a classifier for binding site prediction.

Here, we introduce DyProL, a dynamic protein representation learning framework for protein–nucleic acid binding site prediction. In contrast to conventional single‐structure modeling, DyProL represents each protein as a conformational ensemble sampled from equilibrium‐like structural distributions (Figure 1a), thus capturing structural heterogeneity relevant for nucleic acid recognition. DyProL employs geometry‐aware attention mechanisms that hierarchically integrate intra‐conformation spatial features with inter‐conformation relational patterns. This design enables the model to encode both local geometric determinants of binding and cross‐state structural variability within a unified learning framework. We systematically evaluated DyProL on DNA and RNA‐binding site prediction benchmarks and observed consistent performance gains over static structure–based baselines across multiple datasets. In addition, we conducted a principled investigation of ensemble construction strategies, demonstrating that compact, representative conformational subsets can approximate broader conformational landscapes while enhancing predictive robustness. More generally, our study highlights dynamic ensemble representations as a scalable and biophysically grounded alternative to single‐structure paradigms for structure‐based functional inference.

## 2 Results

### 2.1 Dynamic Protein Representation Learning via Conformational Ensembles (DyProL)

The overall framework of DyProL is illustrated in Figure 1b. Starting from a protein sequence, DyProL first constructs conformational ensembles by large‐scale sampling of equilibrium‐like protein structures using BioEmu [36]. This generative sampling captures a broad range of accessible conformational states and approximates the underlying equilibrium conformational distribution. However, as many sampled structures concentrate near local energy minima, the resulting ensemble typically exhibits significant redundancy. To obtain a compact, yet informative representation of the conformational landscape, DyProL next performs structural alignment to place all sampled conformations within a common coordinate frame, followed by clustering to identify a small set of representative conformations. This procedure preserves the diversity of the ensemble while reducing redundancy, yielding a tractable approximation of the underlying conformational distribution.

The selected representative conformations are then used as input to a multi‐conformation geometric attention module, which learns protein representations directly from the structural ensemble. This geometry‐aware learning framework jointly models spatial features within individual conformations and relational patterns across conformational states. Finally, the learned protein representation is used to predict residue‐level binding probabilities for DNA or RNA in a task‐specific manner.

Building on these representative ensembles, DyProL learns dynamic protein representations using a unified geometry‐aware attention architecture (Figure 1c). The model integrates structural information both within individual conformations and across conformations, enabling the capture of spatial patterns that are conserved, reconfigured, or state‐dependent across the ensemble. By jointly modeling intra‐ and inter‐conformation relationships, DyProL encodes dynamic structural features that are inaccessible to static representations. Detailed descriptions of the workflow are provided in the Methods, with full algorithmic formulations in Algorithms S1 and S2. A detailed description of data collection, redundancy reduction, and dataset splitting is provided in Note S4.

To systematically evaluate the benefit of dynamic protein representation learning, we constructed non‐redundant benchmark datasets covering protein–DNA and protein–RNA binding site prediction tasks. A total of 1278 DNA‐binding proteins (DBPs) and 1166 RNA‐binding proteins (RBPs) were collected, and their length distributions are shown in Figure S1. Redundancy was removed at the protein level to ensure that homologous proteins were not shared between training, validation, or test splits, thereby enabling a rigorous assessment of model generalization. The number of binding and non‐binding sites is listed in Table S1. In addition to the in‐house curated datasets, we further evaluated DyProL on publicly available GraphBind [37] benchmarks to assess cross‐dataset generalization and robustness under independent data curation protocols. The in‐house dataset and the GraphBind benchmark were treated as separate training and evaluation settings. For experiments on the in‐house dataset, all static and dynamic models were trained and evaluated using the same in‐house data splits and the same binding‐site label definition, where a residue was annotated as binding if any of its heavy atoms was within 3.5 Å of any nucleic acid atom. For experiments on the GraphBind benchmark, models were trained using the corresponding GraphBind training set and evaluated on the GraphBind test set following the original GraphBind binding‐site definition.

For each protein, we generated conformational ensembles using BioEmu [36]. Specifically, 100 conformations per protein were sampled and subsequently filtered to remove structures that exhibit severe steric clashes or physically implausible geometries. After filtering, the average number of retained conformations was 84 for DNA‐binding proteins and 83 for RNA‐binding proteins (Figure S2). Across the datasets, the resulting ensembles exhibited substantial structural variability, with mean pairwise Cα RMSD values centered around 5.0–5.5 Å (Figure S3). This range reflects moderate yet functionally relevant conformational deviations, indicating that the sampled ensembles capture meaningful structural heterogeneity beyond trivial rigid‐body fluctuations.

### 2.2 Dynamic Representation Learning Improves Binding Site Prediction by Capturing Conformational Heterogeneity

We designed four evaluation settings to assess the effect of conformational state and training paradigm on binding site prediction. In the first three settings, models were trained on static PDB‐bound structures and evaluated on: (1) the same PDB‐bound state, (2) AlphaFold‐predicted structures, and (3) representative equilibrium conformations derived from ensemble clustering. These configurations isolate the impact of the structural domain shift under static learning. In the fourth setting, DyProL was trained and evaluated on conformational ensembles, enabling explicit modeling of dynamic structural variability. To ensure a fair comparison, the static baseline shares the same network architecture, node features, and edge features as DyProL, but operates on a single static structure per protein, with inter‐conformation message passing disabled. Both dynamic and static models were trained and evaluated on identical data splits.

As summarized in Table 1, the Dynamic model achieved the highest pooled AUPRC and AUROC across all datasets and evaluation settings. However, the gains were generally modest when both training and evaluation were performed using PDB‐bound structures, and the protein‐level sign tests did not reach statistical significance in most datasets under this setting. One possible explanation is that their performance may partially reflect pre‐organized interface features present in bound structures, as binding‐induced geometric patterns are already present in the input structures. As a result, their performance may be partially inflated by pre‐organized interface features, rather than reflecting predictive capability under unbound or conformationally flexible states [38].

**TABLE 1:** Performance comparison across datasets under different protein conformational states and training modes.

Dataset	Training mode	Training states^a^	Testing states^b^	AUPRC	AUROC	BH‐adj. sign‐test q(AUPRC / AUROC)^e^
DBP (In‐house)	Static	PDB‐Bound	PDB‐Bound	0.338	0.860	0.011 / 0.0078
		PDB‐Bound	AlphaFold	0.327	0.858	$3.48\times 10^{-4}$ / $1.42\times 10^{-6}$
		PDB‐Bound	Equi. Repr.^c^	0.326	0.854	$1.14\times 10^{-14}$ / $6.88\times 10^{-14}$
		AlphaFold	AlphaFold	0.332	0.854	0.0041 / $5.35\times 10^{-8}$
	Dynamic	Ensemble^d^	Ensemble	0.353	0.871	– / –
DBP (GraphBind)	Static	PDB‐Bound	PDB‐Bound	0.333	0.863	0.353 / 0.247
		PDB‐Bound	AlphaFold	0.310	0.852	$1.53\times 10^{-5}$ / $5.35\times 10^{-10}$
		PDB‐Bound	Equi. Repr.	0.302	0.848	$8.07\times 10^{-20}$ / $1.12\times 10^{-25}$
		AlphaFold	AlphaFold	0.321	0.856	0.0078 / $1.42\times 10^{-6}$
	Dynamic	Ensemble	Ensemble	0.338	0.870	– / –
RBP (In‐house)	Static	PDB‐Bound	PDB‐Bound	0.331	0.830	0.281 / 0.390
		PDB‐Bound	AlphaFold	0.327	0.828	$6.88\times 10^{-14}$ / $1.96\times 10^{-15}$
		PDB‐Bound	Equi. Repr.	0.326	0.826	$4.68\times 10^{-15}$ / $2.76\times 10^{-16}$
		AlphaFold	AlphaFold	0.331	0.828	0.045 / 0.353
	Dynamic	Ensemble	Ensemble	0.341	0.834	– / –
RBP (GraphBind)	Static	PDB‐Bound	PDB‐Bound	0.205	0.802	0.116 / 0.475
		PDB‐Bound	AlphaFold	0.189	0.796	0.0013 / 0.027
		PDB‐Bound	Equi. Repr.	0.192	0.791	$1.80\times 10^{-11}$ / $8.00\times 10^{-12}$
		AlphaFold	AlphaFold	0.202	0.795	0.0019 / 0.045
	Dynamic	Ensemble	Ensemble	0.212	0.805	– / –

^a^ Training States: protein structural states used during model optimization.

^b^ Testing States: protein structural states used for performance evaluation.

^c^ Equi. Repr.: Equilibrium Representative, the cluster‐center structure derived from conformational ensemble clustering, corresponding to the most populated conformational basin.

^d^ The selected representative conformational ensemble, obtained by clustering BioEmu‐sampled conformations and jointly used for model training or evaluation.

^e^ One‐sided protein‐level paired sign test comparing Dynamic with each Static baseline; values are BH‐adjusted q‐values for per‐protein AUPRC/AUROC.

When evaluated on AlphaFold‐predicted structures, static models generally showed reduced performance compared with their PDB‐bound evaluation setting (Table 1). This setting is practically important because AlphaFold‐predicted structures are often the most accessible inputs for binding site prediction, whereas experimentally resolved bound complexes are frequently unavailable [39]. Under AlphaFold‐based evaluation, DyProL showed more pronounced and generally significant improvements over the corresponding static baselines. Compared with the best‐performing static baseline trained and evaluated on AlphaFold structures, DyProL increased AUPRC by 6.3% for DNA‐binding proteins and 3.0% for RNA‐binding proteins on the in‐house dataset, and by 5.3% and 5.0%, respectively, on the GraphBind dataset. These results highlight the practical advantage of dynamic representations for binding site prediction under realistic structural conditions.

When evaluated on equilibrium representative conformations, static models generally showed further performance degradation compared with evaluation on AlphaFold‐predicted structures (Table 1). This suggests that conformational changes can substantially affect the performance of static models, particularly when a single representative structure is used to approximate a heterogeneous conformational landscape. To further examine this effect, we curated a set of 15 DNA‐binding proteins from Ref. [19], for which both bound and unbound conformations are experimentally resolved in the PDB and which exhibit substantial structural differences between the two states (TM‐score <0.85). For each protein, we compared the AlphaFold‐predicted structure, the BioEmu‐generated ensemble, and the BioEmu equilibrium representative conformation with the corresponding bound and unbound reference structures. RMSDs were computed using common Cα atoms after optimal rigid‐body superposition.

**FIGURE 2:**
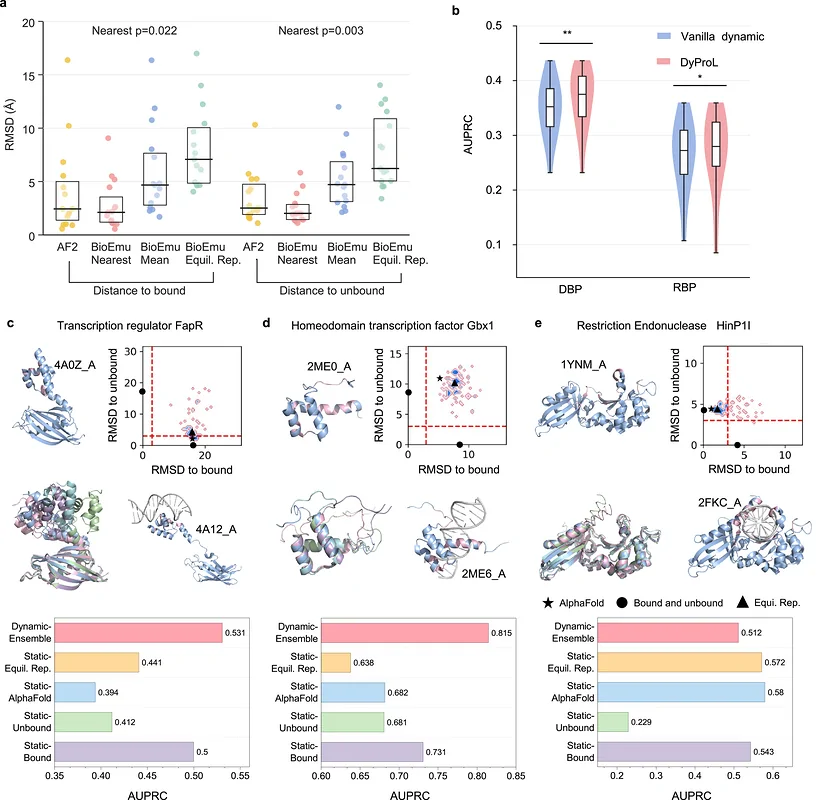
Dynamic conformational ensembles improve nucleic acid binding site prediction. (a) RMSD distributions of AlphaFold2 structures, nearest BioEmu conformations, mean BioEmu ensemble RMSDs, and BioEmu cluster representative conformations relative to experimentally resolved bound and unbound reference structures. Each point represents one of the 15 paired DNA‐binding proteins. RMSDs were computed using common Cα atoms after optimal rigid‐body superposition. P values indicate paired Wilcoxon signed‐rank tests comparing AlphaFold2 RMSD with the nearest BioEmu RMSD. **(b**) Comparison between the vanilla dynamic baseline and DyProL on DBP and RBP datasets, showing that DyProL achieves significantly higher AUPRC than simple ensemble‐averaged predictions from a static model. **(c–e**) Representative examples demonstrating the impact of conformational heterogeneity on binding site prediction. Sampled conformations are visualized as superimposed structures (middle left), and their distributions are quantified by RMSD to bound and unbound reference states (top right). Each point corresponds to a sampled conformation; dashed red lines indicate reference thresholds (3 Å). The star marks the AlphaFold‐predicted structure, circles denote bound and unbound conformations, and triangles indicate the equilibrium representative (Equi. Rep.). Panels show (c) FapR, (d) Gbx1, and (e) HinP1I, with nucleic acid–bound complexes provided for reference. Bar plots report AUPRC for different structural representations. Performance of static models varies with conformational proximity to the bound state, whereas dynamic ensemble representations consistently achieve higher accuracy. These results highlight the importance of capturing conformational heterogeneity for robust binding site prediction.

As shown in Figure 2a, the nearest BioEmu conformation was significantly closer than the AlphaFold2‐predicted structure to both the bound reference and the unbound reference. Specifically, the median RMSD to the bound state was reduced from 2.437 Å for AlphaFold2 to 2.115 Å for the nearest BioEmu conformation (paired Wilcoxon test, p=0.022), while the median RMSD to the unbound state was reduced from 2.516 to 2.021 Å (p=0.003). We further quantified the coverage ratio of structures close to the bound and unbound references across RMSD thresholds from 1 to 5 Å (Figure S5). BioEmu ensembles showed higher coverage than AlphaFold2 for both reference states at most thresholds. For example, at the 3 Å threshold, BioEmu covered bound‐like conformations for 11 of 15 proteins and unbound‐like conformations for 12 of 15 proteins, compared with 9 of 15 proteins for AlphaFold2 in both cases. In contrast, the equilibrium representative conformation was often farther from either endpoint, indicating that a single representative structure may not adequately capture the conformational states relevant to binding. These results indicate that BioEmu samples heterogeneous bound‐like, unbound‐like, and intermediate conformations, rather than simply collapsing onto the bound state. This broader conformational coverage provides a mechanistic explanation for why dynamic representations are more robust than static representations when proteins are evaluated under realistic, flexible structural conditions.

To elucidate the structural basis underlying this behavior, we visualize three representative DNA‐binding proteins: FapR, Gbx1, and HinP1I, all of which undergo pronounced conformational changes between bound and unbound states. Comparative analysis reveals that prediction performance of static models strongly depends on the structural proximity of the input conformation to the bound state. When AlphaFold‐predicted or representative conformations are distant from the bound state, prediction accuracy deteriorates markedly (Figure 2c,d). Conversely, when these conformations are closer to the bound state, performance approaches that achieved using bound structures (Figure 2e), indicating that static models rely heavily on pre‐organized binding geometries. By contrast, DyProL leverages ensemble‐based representations to integrate complementary structural information across multiple conformations, enabling the identification of binding‐competent geometries even when they are absent in individual structures. Collectively, these observations indicate that single‐structure representations are inherently sensitive to conformational selection, whereas ensemble‐based modeling provides a more stable and comprehensive characterization of binding‐relevant structural features.

We further examined whether DyProL performance relies on BioEmu‐generated conformations that closely resemble experimentally determined bound states. We performed a bound‐like conformation exclusion analysis, in which all BioEmu conformations within 3 ÅRMSD of the experimentally determined bound structures were removed before clustering and downstream prediction, without retraining DyProL. After excluding these bound‐like conformations, DyProL showed highly consistent performance, with only a modest relative decrease in AUPRC of 0.8% for DBPs and 0.9% for RBPs. These results indicate that the predictive advantage of DyProL does not primarily originate from direct access to bound‐like conformations, but rather from its ability to integrate heterogeneous conformational information across multiple states.

To evaluate whether the benefit of DyProL could be explained by simple use of multiple conformations, we constructed a vanilla dynamic baseline based on test‐time ensemble averaging. A static model trained on PDB‐bound structures was applied independently to the 12 BioEmu‐generated representative conformations of each protein, and the residue‐level binding probabilities were averaged across conformations. As shown in Figure 2b, this vanilla dynamic strategy provided a useful but limited dynamic baseline, whereas DyProL achieved consistently higher AUPRC on both DBP and RBP datasets, with statistically significant improvements. These results demonstrate that simply exposing a static predictor to multiple conformations is insufficient to reproduce the full benefit of DyProL. The superior performance of DyProL supports the importance of the proposed multi‐conformation geometric attention architecture, which explicitly learns residue‐level relationships across conformations rather than treating conformations as independent predictions to be averaged. Thus, the performance gain of DyProL reflects not only the availability of conformational diversity, but also the ability of the architecture to learn coherent ensemble‐aware representations.

### 2.3 Determining the Optimal Size of Conformational Ensembles

A central component of DyProL is the construction of a representative conformational ensemble that captures the structural variability of the protein while remaining computationally tractable. In the DyProL framework, ensemble construction consists of two stages: (1) large‐scale sampling of equilibrium conformations using BioEmu, and (2) selection of a compact set of representative conformations via clustering. This two‐stage strategy is motivated by the observation that densely sampled ensembles are often highly redundant around local energy minima. Here, we systematically evaluate both the sufficiency of large‐scale sampling and the compactness of the resulting representative ensembles.

In the main benchmarks, BioEmu was used to generate 100 conformations per protein for both training and evaluation. This number was selected to balance computational cost and conformational diversity. To assess whether 100 conformations sufficiently approximate the broader conformational landscape, we conducted an additional large‐scale sampling experiment. Specifically, 50 proteins were randomly selected from the DNA‐bound‐unbound dataset of GeoBind [19], and both 100 and 1,000 conformations per protein were generated using BioEmu.

**FIGURE 3:**
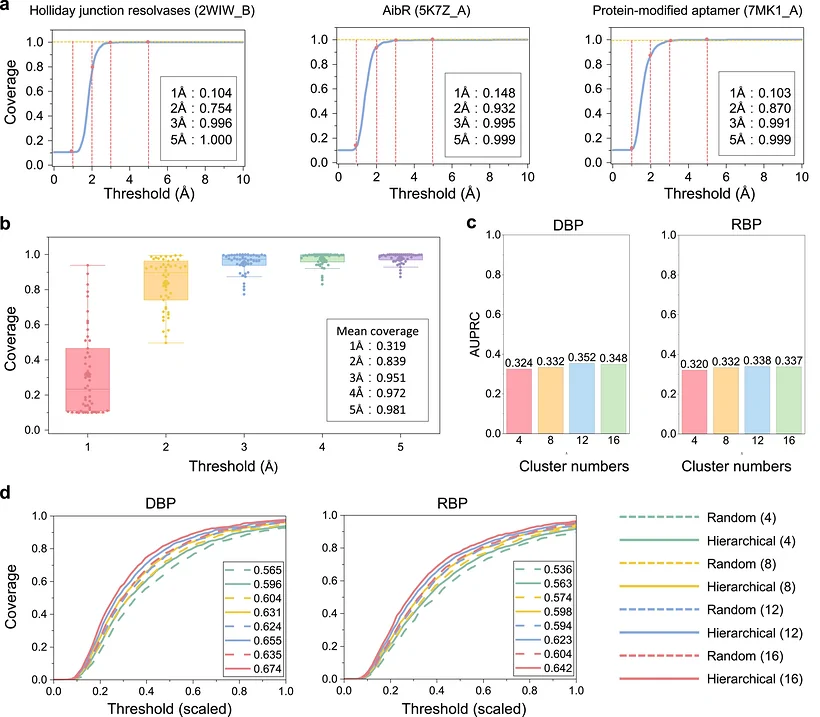
Evaluation of conformational ensemble coverage and clustering strategies. (a) Coverage of 100‐sample ensembles relative to 1000‐sample ensembles across RMSD thresholds for representative proteins (2WIW_B, 5K7Z_A and 7MK1_A). Each point indicates the fraction of 1,000 sampled conformations whose minimum Cα RMSD to any structure in the 100‐conformation ensemble falls below the specified threshold. (b) Distribution of coverage rates across proteins from the DNA‐bound‐unbound dataset of GeoBind, evaluated at different RMSD thresholds. (c) Binding site prediction performance (AUPRC) as a function of the number of clusters selected by hierarchical clustering for DBP and RBP datasets. (d) Coverage curves of representative conformations selected by hierarchical clustering compared with randomly selected conformations of equal size. Coverage is computed relative to the full sampled ensemble across scaled RMSD thresholds (threshold divided by 10). The boxed values indicate the Area under the Coverage Curve (AUCC), which summarizes ensemble representativeness across structural tolerances.

We quantified ensemble sufficiency using a *coverage rate* metric. Given an RMSD threshold τ, coverage at threshold τ is defined as the fraction of conformations in the 1000‐sample ensemble whose minimum Cα RMSD to any structure in the 100‐conformation ensemble is smaller than τ (see Methods for details). Formally, this measures how well the smaller ensemble approximates the conformational support of the larger one under structural tolerance τ. All 50 proteins exhibited high coverage at an RMSD threshold of 5 Å (Figure 3a and Figure S4). As shown in Figure 3b, the 100‐conformation ensembles achieved a mean coverage rate of 95.1% at 3 Å, increasing to 98.1% at 5 Å. These results indicate that 100 conformations provide a high‐resolution approximation of the conformational landscape sampled by BioEmu. Importantly, this sufficiently large ensemble serves as a stable structural basis from which a smaller set of representative conformations can be reliably selected.

While larger ensembles improve coverage of the conformational landscape, they also introduce substantial redundancy and increased computational cost. To establish a principled trade‐off between ensemble size and representational completeness, we further analyzed the impact of clustering on ensemble representativeness. For hierarchical clustering, we introduce the *Area under the Coverage Curve* (AUCC) as a summary metric (see Methods). Coverage rates are computed across a range of RMSD thresholds, yielding a coverage curve in RMSD–coverage space (Figure 3d). The AUCC, defined as the area under this curve, provides an aggregate measure of ensemble representativeness across both fine‐grained (small τ) and coarse‐grained (large τ) structural tolerances. Higher AUCC values therefore reflect not only broader coverage of the conformational landscape but also improved structural fidelity under more stringent similarity thresholds. As expected, AUCC increases monotonically with the number of representative conformations; however, the marginal gains progressively diminish, indicating increasing redundancy within the ensemble.

To directly connect ensemble representativeness with predictive utility, we trained DyProL using varying numbers of representative conformations selected by hierarchical clustering and evaluated binding site prediction performance. As shown in Figure 3c, predictive performance improved steadily as the number of representative conformations increased, reaching a maximum at 12 conformations. Beyond this point, performance plateaued or slightly declined, suggesting that additional conformations contribute limited novel structural information while increasing model complexity and potential noise. These results indicate that a moderate level of structural diversity is optimal for learning functionally relevant dynamic features.

In addition, we compared hierarchical clustering with alternative clustering strategies, including DBSCAN. Although DBSCAN produced reasonable representative ensembles, models trained with DBSCAN‐selected conformations consistently underperformed those based on hierarchical clustering (Figure S6), likely due to less stable control over cluster granularity and representative selection.

Together, these analyses demonstrate that DyProL achieves an effective balance between conformational coverage and computational efficiency. A sufficiently large sampled ensemble enables reliable approximation of the underlying conformational landscape, while a carefully selected small set of representative conformations maximizes downstream predictive performance. This two‐stage design provides both structural completeness and computational tractability.

### 2.4 Quality Assessment of Conformational Ensembles

**FIGURE 4:**
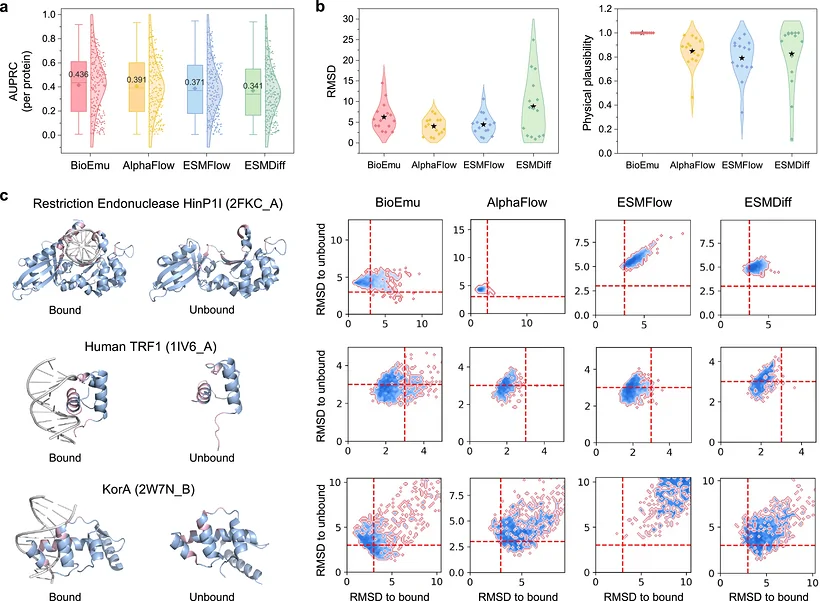
Comparison of conformational ensembles generated by four protein generative models. (a) Binding site prediction performance on the DBP dataset using conformational ensembles generated by BioEmu, AlphaFlow, ESMFlow, and ESMDiff. AUPRC is computed per protein, and values indicate the median across proteins. (b) Structural diversity and physical plausibility of the generated ensembles. 15 proteins that exhibit pronounced structural differences between bound and unbound states are analyzed. For each method, the mean pairwise Cα RMSD among representative conformations (left) and the physical plausibility rate (right) are shown. Statistical comparisons of the metrics shown in Figure 4b are provided in Tables S4 and S5, with proteins treated as paired experimental units across the four ensemble‐generation methods. (c) Representative conformational landscapes for proteins 2FKC_A, 1IV6_A, and 2W7N_B. Experimentally determined bound and unbound structures are shown (left), followed by two‐dimensional RMSD maps of conformations relative to the bound (*x*‐axis) and unbound (*y*‐axis) states for each method. Dashed lines indicate RMSD thresholds (3 Å) separating bound‐like and unbound‐like regions.

Accurately approximating dynamic proteins using multi‐conformation ensembles requires the selection of ensembles that are both structurally diverse and physically plausible, as ensemble quality directly influences the predictive performance of dynamic binding site models. Existing approaches for ensemble generation include molecular dynamics (MD) simulations and generative model–based methods. While MD simulations provide physically grounded trajectories, they are computationally expensive and often insufficient for exhaustively exploring conformational landscapes within practical timescales. Therefore, we evaluated four generative approaches–BioEmu [36], AlphaFlow [34], ESMFlow [34], and ESMDiff [35]–for ensemble construction and systematically compared their downstream impact on model performance using the DBP dataset. As shown in Figure 4a and Figure S7, BioEmu‐generated ensembles achieved the highest median AUPRC across proteins, followed by AlphaFlow and ESMFlow, whereas ESMDiff yielded the lowest performance, indicating that ensemble quality substantially affects predictive accuracy.

To further investigate the origin of these differences, we analyzed ensemble diversity and structural plausibility across the four sampling methods. We curated a set of 15 DBPs from Ref. [19], for which both bound and unbound conformations are experimentally resolved in the PDB and which exhibit substantial structural differences (TM‐score < 0.85), enabling a controlled assessment of conformational coverage and sampling behavior.

We first calculated pairwise Cα RMSD distributions and physical plausibility scores. These metrics quantify the trade‐off between structural diversity and physical realism, two key factors expected to influence downstream prediction performance.

Figure 4b shows that BioEmu generates ensembles with broad structural diversity while maintaining high physical realism. To quantitatively support this comparison, we further performed paired non‐parametric statistical analyses across the same 15 proteins (Tables S4 and S5). The four methods differed significantly in mean pairwise Cα RMSD (Friedman $\chi^{2}=15.08$, p=0.00175, Kendall's W=0.335; Supplementary Table S4) and physical plausibility rate (Friedman $\chi^{2}=30.27$, $p=1.21\times 10^{-6}$, Kendall's W=0.673; Table S4). Post‐hoc paired Wilcoxon signed‐rank tests with Holm correction showed that BioEmu produced significantly higher mean pairwise Cα RMSD than AlphaFlow (adjusted p=0.00256) and ESMFlow (adjusted p=0.0418), whereas its RMSD was not significantly different from ESMDiff (adjusted p=0.375; Table S5). In contrast, BioEmu showed significantly higher physical plausibility than AlphaFlow, ESMFlow, and ESMDiff (adjusted p=0.000366, 0.00327, and 0.00887, respectively; Table S5). These results indicate that BioEmu provides a favorable balance between conformational diversity and physical realism, whereas the larger RMSD values observed for ESMDiff are accompanied by lower physical plausibility.

A more detailed analysis of two‐dimensional (2D) RMSD landscapes relative to bound and unbound reference structures (Figure 4c and Figures S8–S10) reveals systematic differences in sampling behavior between methods. For 2FKC_A (Figure 4c upper row), BioEmu‐generated ensembles span bound, unbound, and intermediate conformations, forming a distribution consistent with equilibrium‐like exploration of the conformational landscape. In contrast, AlphaFlow predominantly samples bound‐like conformations with minimal coverage of the unbound basin. ESMFlow and ESMDiff generate conformations that are displaced from both bound and unbound states, indicating sampling outside major functional conformational regions. For 1IV6_A (Figure 4c middle row), AlphaFlow, ESMFlow, and ESMDiff all cluster primarily around the bound state, with limited exploration of unbound conformations, whereas BioEmu captures a broader distribution spanning both states. For 2W7N_B (Figure 4c bottom row), all methods exhibit partially equilibrium‐like distributions; however, ESMFlow conformations display reduced structural fidelity and increased dispersion away from both reference basins, suggesting lower ensemble quality despite apparent diversity.

In addition to evaluating generative‐model‐based ensemble construction strategies, we examined whether DyProL could generalize to conformational ensembles derived from experimentally determined apo structures and physics‐based simulations. For each protein in the 256‐protein in‐house DBP test set, we searched for homologous apo structures determined by NMR and retained matches with sequence identity greater than or equal to 90%. Suitable apo NMR counterparts were identified for 12 test proteins. Residue‐level binding annotations from the corresponding DNA‐bound structures were transferred to the apo NMR sequences through pairwise sequence alignment. The deposited NMR conformers were treated as experimentally derived apo ensembles, and an additional 20‐ns MD simulation was performed for each target starting from the first NMR conformer. BioEmu ensembles showed the greatest structural dispersion, followed by MD‐derived ensembles, whereas NMR ensembles were the most compact (Figure S11a). DyProL was trained on BioEmu ensembles and directly evaluated on the three ensemble sources. BioEmu achieved the highest AUPRC of 0.331, followed by MD at 0.312 and NMR at 0.303 (Figure S11b). These results indicate that DyProL can generalize to physically grounded apo‐state ensembles, although the higher BioEmu performance may partly reflect its broader conformational coverage and the smaller train–test distribution shift.

Collectively, these results indicate that BioEmu provides the most favorable empirical trade‐off between structural diversity, physical plausibility, and downstream predictive performance among the evaluated methods. However, BioEmu does not uniformly recover all bound, unbound, and intermediate conformational basins for every protein. We therefore interpret BioEmu‐generated ensembles as approximate sequence‐conditioned conformational hypotheses, rather than complete reconstructions of the functional conformational landscape.

### 2.5 Evaluation of Dynamic Representations in IDR‐like/Flexible Regions

To clarify which regions benefit most from conformationally dynamic representation learning, we stratified residues in the DyProL DBP and RBP test sets according to DSSP [40] secondary‐structure annotations. Residues in regular secondary structures (H/G/I/E/B) were classified as non‐IDR‐like regions, whereas residues in turns, bends, or non‐regular coil states (T/S/blank) were classified as IDR‐like/flexible regions. We use the term “IDR‐like/flexible” because DSSP describes the local structural state in a modeled conformation rather than intrinsic disorder propensity.

**FIGURE 5:**
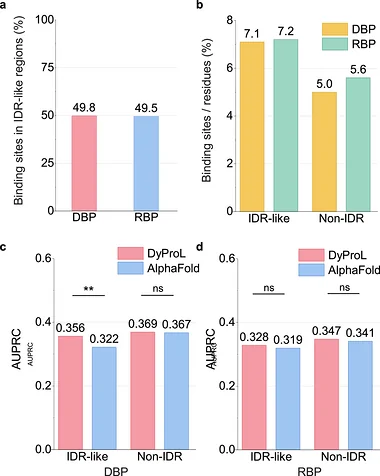
Evaluation of dynamic representations in IDR‐like/flexible regions. (a) Proportion of annotated binding sites located in IDR‐like/flexible regions in the DBP and RBP test sets. (b) Binding‐site density in IDR‐like and non‐IDR‐like regions, calculated as the percentage of binding residues among all residues in each structural category. (c,d) AUPRC comparison between DyProL and the static AlphaFold‐based model in IDR‐like/flexible and non‐IDR‐like regions for c DBPs and d RBPs. ** indicates BH‐adjusted q<0.01; ns indicates not significant.

This analysis showed that nucleic acid‐binding residues are frequently located in flexible structural contexts. Approximately half of all annotated binding sites were found in IDR‐like/flexible regions for both DBPs and RBPs, with proportions of 49.8% and 49.5%, respectively (Figure 5a). After normalization by the number of residues in each structural category, IDR‐like/flexible regions also showed higher binding‐site density than non‐IDR‐like regions. For DBPs, 7.1% of IDR‐like/flexible residues were annotated as binding sites, compared with 5.0% of non‐IDR‐like residues; for RBPs, the corresponding values were 7.2% and 5.6%, respectively (Figure 5b). These results indicate that flexible regions constitute an important component of protein–nucleic acid binding interfaces and are enriched for binding residues relative to more regular structural regions.

We next compared DyProL with the static AlphaFold‐based model within each structural category. For DBPs, DyProL achieved a higher AUPRC than the static model in IDR‐like/flexible regions (0.356 vs. 0.322), whereas the two models performed similarly in non‐IDR‐like regions (0.369 vs. 0.367; Figure 5c). For RBPs, DyProL showed a modest numerical improvement over the static model in both IDR‐like/flexible regions (0.328 vs. 0.319) and non‐IDR‐like regions (0.347 vs. 0.341), but neither difference reached statistical significance (Figure 5d). Thus, the most evident improvement was observed in IDR‐like/flexible regions of DBPs, where the dynamic ensemble representation significantly outperformed the static AlphaFold‐based model. These results suggest that conformationally dynamic representations can provide additional benefits in flexible structural regions, although the magnitude and statistical significance of this effect may vary between DNA‐ and RNA‐binding proteins.

### 2.6 Comparison with Existing Methods

DyProL is a general framework that enables flexible integration of heterogeneous information into node‐level representations. Given the established importance of sequence‐derived evolutionary information for nucleic acid binding site prediction, we augment DyProL with both multiple sequence alignment (MSA) features and protein language model (PLM) embeddings (see Note S3 for details).

We evaluated DyProL on the benchmark datasets curated by GraphBind [37], which are widely adopted for nucleic acid binding site prediction, and compared its performance with four recent state‐of‐the‐art methods, namely EquiPNAS [4], GraphSite [39], GeoNet [41], and GraphBind [37]. To better reflect realistic application scenarios, all methods were trained and evaluated using AlphaFold‐predicted protein structures, under the assumption that experimentally determined nucleic acid–bound conformations are unavailable.

**FIGURE 6:**
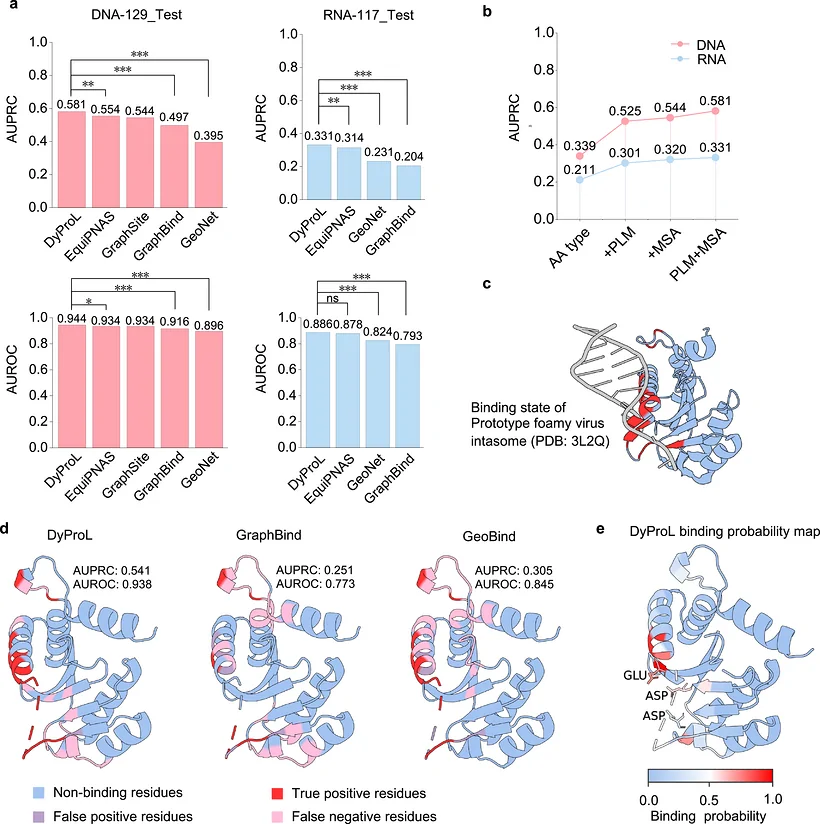
Comparison of DyProL with existing methods on GraphBind benchmark datasets and a representative case study. (a) Performance comparison on the GraphBind benchmark test sets (DNA‐129 and RNA‐117). DyProL achieves the best performance across both datasets, as measured by AUPRC and AUROC. *, ** and *** indicate Benjamini–Hochberg adjusted sign‐test q‐values of q<0.05, q<0.01 and q<0.001, respectively. ns indicates not significant. (b) Feature ablation of DyProL on the GraphBind benchmark datasets. AUPRC was compared using amino‐acid type alone, with PLM embeddings, with MSA features, and with both PLM and MSA features on DNA‐129 and RNA‐117 dataset. (c) Structure of the prototype foamy virus (PFV) intasome (PDB: 3L2Q), a retroviral integrase structurally related to HIV‐1 integrase. Chain A of 3L2Q serves as a structural reference for the integrase fold corresponding to 1BIS_A. (d) Predicted binding sites on 1BIS_A generated by three representative methods: DyProL, GraphBind, and GeoBind. Compared to baseline methods, DyProL produces more spatially coherent predictions and better recovers functionally relevant regions. (e) Residue‐level prediction scores produced by DyProL, visualized on the structure of 1BIS_A using a blue‐to‐red color gradient, where warmer colors indicate higher predicted binding likelihood. DyProL highlights the conserved catalytic triad (Asp64, Asp116, and Glu152), indicating its ability to capture functionally relevant regions associated with ligand recognition and catalytic activity.

As shown in Figure 6a, DyProL consistently achieves the best performance across both DNA and RNA test sets, outperforming all competing methods in terms of both AUPRC and AUROC. On the DNA‐129 test set, DyProL attains an AUPRC of 0.581, representing a relative improvement of 4.8% over the strongest baseline (EquiPNAS, 0.554), and a gain of 6.8% compared to GraphSite (0.544). On the RNA‐117 test set, DyProL achieves an AUPRC of 0.331, improving upon the strongest baseline (EquiPNAS, 0.314) by 5.4%. Importantly, these consistent gains across both metrics and datasets cannot be attributed solely to enhanced feature representations, as competing methods already leverage rich sequence‐ and structure‐derived information. Instead, they highlight the advantage of explicitly modeling structural variability through conformational ensembles, enabling DyProL to capture functionally relevant conformational heterogeneity beyond conventional static representations.

We further performed a feature ablation analysis to quantify the contribution of different input features. We compared four feature settings: amino‐acid type alone, amino‐acid type plus PLM embeddings, amino‐acid type plus MSA features, and the combination of PLM and MSA features. Starting from amino‐acid type alone, the incorporation of PLM embeddings and MSA features improved AUPRC on the DNA‐129_Test and RNA‐117_Test benchmark (Figure 6b). The largest gain was observed after adding MSA‐derived features, indicating that evolutionary information remains highly informative for nucleic acid binding‐site prediction. Nevertheless, DyProL achieved its best performance when both PLM and MSA features were combined, suggesting that learned sequence representations and evolutionary profiles provide complementary information.

To further assess performance in challenging binding scenarios, we conducted a case study on 1BIS_A (HIV‐1 integrase), for which no experimentally determined binding‐state structure with an identical sequence is available. However, structurally related retroviral integrases, such as the prototype foamy virus (PFV) intasome (PDB: 3L2Q), share a highly similar fold despite sequence divergence (Figure 6c and Figure S12). The bound structure of 3L2Q_A therefore provides a structural reference for identifying functionally relevant regions, while 1BIS_A represents a realistic apo‐like test case.

We compared DyProL with GeoBind [19] and GraphBind [37]. As shown in Figure 6d, baseline predictions exhibit fragmented spatial coverage and miss several binding‐relevant regions, leading to both false negatives and spurious predictions. In contrast, DyProL produces more spatially coherent predictions and better recovers major binding regions, improving AUPRC by 0.236 and AUROC by 0.093 over the best baseline (GeoBind). Visualization of residue‐level confidence scores (Figure 6e) shows that residues near the inferred binding interface consistently receive higher scores, indicating that DyProL learns spatially consistent decision patterns aligned with structural organization. Interestingly, DyProL also highlights residues within the conserved catalytic triad of 1BIS_A (Asp64, Asp116, and Glu152), even though not all of them are annotated as direct binding sites. This observation suggests that DyProL captures functionally relevant structural coupling beyond immediate geometric proximity.

Taken together, these results demonstrate that DyProL improves binding site prediction on benchmark datasets and generalizes effectively to challenging scenarios where binding‐competent conformations are not directly observed.

## 3 Conclusion

Protein function is inherently dynamic, yet most structure‐based learning frameworks reduce this complexity to a single static conformation [22, 25]. Our results demonstrate that this paradigm is fundamentally limiting for nucleic acid binding site prediction, where functional recognition often depends on conformational transitions that are not captured in experimentally resolved or predicted structures [23, 42]. By explicitly modeling conformational ensembles, DyProL addresses this limitation and consistently improves predictive performance across diverse evaluation settings.

The performance gains arise not from simple aggregation of multiple structures, but from learning coherent representations across conformations while preserving residue‐level correspondence. This enables the model to disentangle conserved structural features from state‐specific variations and to capture binding‐relevant geometries that may not be present in any individual structure. Such capability is particularly important for protein–nucleic acid interactions, where binding frequently involves conformational selection or induced‐fit mechanisms [43]. In this sense, DyProL represents a shift from structure‐based learning toward ensemble‐based representation learning, reframing protein modeling as learning over distributions of conformational states rather than single structural snapshots [44].

We note that DyProL is designed to represent conformational variability across sampled structural ensembles rather than to explicitly model protein dynamics in a kinetic or time‐resolved manner. Accordingly, it does not aim to resolve transition pathways or infer state populations with physical rigor. Instead, the generated conformations are intended to approximate an equilibrium‐like structural distribution [36], providing a coarse but functionally relevant description of conformational variability. Despite this simplification, such an approximation is sufficient for binding‐site prediction, where the primary requirement is to capture the diversity of binding‐competent geometries rather than their temporal ordering. Importantly, this design also ensures computational tractability, enabling application to large‐scale datasets. Notably, the advantages of this approach are most evident in realistic scenarios where only apo or predicted structures (e.g., AlphaFold) are available [39], which is common for many nucleic acid binding proteins lacking experimentally resolved complexes. This highlights the practical utility of DyProL under structural uncertainty.

More broadly, DyProL is a general framework for protein representation learning. This perspective may extend to a wide range of structure–function learning problems, including ligand binding, allosteric regulation, and conformational state prediction, as well as broader classes of protein–nucleic acid recognition processes. Nevertheless, several limitations remain. The quality of the conformational ensemble depends on the underlying generative model and may include physically implausible or functionally irrelevant states. Current clustering strategies are based primarily on geometric similarity and may not fully align with functional distinctions, and all conformations are treated equally without accounting for their relative populations. Addressing these challenges through improved generative modeling, function‐aware clustering, and weighted representations represents an important direction for future work. In summary, our study identifies a fundamental limitation of single‐structure paradigms and establishes dynamic ensemble modeling as a principled framework for protein representation learning under structural uncertainty.

## 4 Methods

### 4.1 Data Curation

DNA‐ and RNA‐binding protein structures were collected from the Nucleic Acid Knowledgebase (NAKB) [45]. Only experimentally resolved structures with a resolution better than 3.5 Å were retained. To reduce sequence redundancy before dataset splitting, protein chains were clustered using CD‐HIT [46] with progressively decreasing sequence identity thresholds, followed by a final clustering threshold of 0.3. Proteins longer than 1000 amino acids were removed to ensure tractable structure prediction and conformational ensemble generation. The resulting non‐redundant DBP and RBP datasets were then split into training, validation, and test sets, ensuring that proteins from the same sequence cluster were not assigned to different subsets. A detailed description of data collection, redundancy reduction, length filtering, dataset splitting, and train–test homology verification is provided in Note S4.

To further assess potential train–test sequence leakage, each test‐set protein was compared against all training‐set proteins using BLASTP. The maximum pairwise sequence identity between each test‐set sequence and its closest train‐set match was reported in Tables S2 and S3, confirming that no train–test protein pair exceeded the 40% sequence identity threshold. The distribution of protein sequence lengths is shown in Figure S1. Residue‐level binding labels were defined using a distance‐based criterion: a residue was annotated as a binding site if any of its heavy atoms was within 3.5 Å of any nucleic acid atom. A summary of dataset statistics is provided in Table S1.

### 4.2 Protein Conformational Ensemble Generation and Selection

Protein conformational ensembles were generated using BioEmu [36], a frozen sequence‐conditioned generative model that takes only the amino‐acid sequence as input. No binding‐site labels, nucleic acid coordinates, experimentally determined target bound complexes, or benchmark split information were used during ensemble generation; thus, the generated structures were treated as sequence‐conditioned conformational hypotheses rather than sources of supervised binding‐site information.

For each protein, 100 conformations were sampled. Conformations that violated basic physical constraints were discarded, resulting in an average of more than 70 valid conformations per protein. To reduce computational cost and remove redundant conformations, we performed clustering on the sampled ensembles and selected representative conformations for downstream analysis.

Each conformation was encoded using frame‐based geometric features derived from backbone atoms. Specifically, rotation‐invariant Cα–Cα distances at multiple sequence separations, together with backbone dihedral angles (ϕ, ψ) represented by their sine and cosine values, were extracted to form a compact structural descriptor. Based on these representations, clustering was performed using a hierarchical clustering method. From each ensemble, 12 representative conformations were selected from the resulting clusters and used for subsequent multi‐conformation learning.

For the selected representative conformations, pairwise structural alignment was first performed to compute the RMSD between all conformation pairs, resulting in a pairwise RMSD distance matrix. Based on this matrix, the conformation with the smallest average RMSD to all others was selected as the reference structure. All remaining conformations were then rigidly aligned to this reference, ensuring a consistent spatial coordinate system across the ensemble. This alignment procedure enables the subsequent multi‐conformation learning model to capture meaningful geometric relationships between conformations, as structural variations are disentangled from arbitrary global rotations and translations.

### 4.3 Multi‐Conformation Learning Neural Network

#### 4.3.1 Multi‐Conformation Graph Representation

For each protein, we represent its conformational ensemble as a *multi‐conformation graph*. Given a protein sequence of length N, we consider an ensemble of K conformations sampled from an equilibrium distribution. All conformations share the same residue identity and sequence order, while differing in their 3D geometry.

Formally, the ensemble is defined as

(1) $$E=\{G^{\left(k\right)}|k=1,\cdots ,K\},$$

where each conformation graph $G^{\left(k\right)}=\left(V,E^{\left(k\right)}\right)$ contains a common node set

(2) $$V=\{v_{1},v_{2},\cdots ,v_{N}\},$$

corresponding to amino acid residues, and a conformation‐specific edge set $E^{\left(k\right)}$ defined by spatial proximity.

Each node $v_{i}^{\left(k\right)}$ is associated with (1) a residue feature embedding derived from amino acid identity (and optionally pretrained language model features), (2) a Cartesian coordinate $x_{i}^{\left(k\right)}\in R^{3}$, and (iii) a local reference frame $R_{i}^{\left(k\right)}\in R^{3\times 3}$. $R_{i}^{\left(k\right)}$ is a rotation‐equivariant orthonormal coordinate system $\left[n_{i},u_{i},v_{i}\right]$, built on local orientations between N, Cα, and C [47] describing residue‐level orientation. Within each conformation $G^{\left(k\right)}$, spatial neighbors $E^{\left(k\right)}$ are constructed by selecting the M closest residues in Euclidean space, resulting in a local geometric graph.

#### 4.3.2 Geometric Attention with Local Coordinate Encoding

Both structure‐wise and ensemble‐wise message passing are implemented using the same *geometric attention* module, allowing spatial information to be processed in a unified manner across the two aggregation schemes.

For a residue pair (i,j), the geometric relations are encoded in the local coordinate frame of residue i through a relative position term and a relative orientation term. Specifically, let $x_{i}\in R^{3}$ denote the Cartesian coordinate of residue i, and let $\left(n_{i},u_{i},v_{i}\right)$ define its local orthonormal basis. The relative position of residue j with respect to residue i is first projected onto the local frame of residue i:

(3) $$\begin{array}{c} P_{ij}={\left(x_{j}-x_{i}\right)}^{T}\left[n_{i}\;|\;u_{i}\;|\;v_{i}\right], \end{array}$$

where $P_{ij}\in R^{3}$. The relative orientation is represented by expressing the local basis of residue j in the coordinate system of residue i:

(4) $$\begin{array}{c} O_{ij}=\left[\begin{array}{c} n_{j}^{T}\\ u_{j}^{T}\\ v_{j}^{T} \end{array}\right]\left[n_{i}\;|\;u_{i}\;|\;v_{i}\right], \end{array}$$

where $O_{ij}\in R^{3\times 3}$. The final pairwise geometric descriptor is defined as

(5) $$\begin{array}{c} R_{ij}=\left[\;P_{ij}∥\mathrm{vec}\left(O_{ij}\right)\;\right]\in R^{12}, \end{array}$$

where ∥ denotes concatenation and vec(·) flattens the orientation matrix into a nine‐dimensional vector.

Let $h_{i}^{\ell }$ denote the hidden representation of residue i at the ℓ‐th geometric transformer block. Before attention is computed, the representation of each neighboring residue j is modulated by its pairwise geometric relation to residue i:

(6) $$\begin{array}{c} {\tilde{h}}_{j|i}^{\;\ell }=w_{ij}\;\phi_{g}^{\ell }\left(R_{ij}\right)⊙h_{j}^{\ell }, \end{array}$$

where

(7) $$\begin{array}{c} w_{ij}=\exp\;\left(-\frac{∥x_{i}-x_{j}{∥}_{2}^{2}}{2\sigma^{2}}\right) \end{array}$$

is a Gaussian distance kernel, $\phi_{g}^{\ell }\left(\cdot \right)$ is a learnable geometric encoder implemented as an MLP, and ⊙ denotes element‐wise modulation. In this way, the residue feature is adaptively reweighted according to both spatial proximity and relative geometry.

The geometric attention layer then computes queries from the central residue and keys and values from the geometry‐conditioned neighbor features:

(8) $$\begin{array}{c} Q_{i}^{\ell }=Q^{\ell }\left(h_{i}^{\ell }\right),\;K_{j|i}^{\ell }=K^{\ell }\left({\tilde{h}}_{j|i}^{\;\ell }\right),\;V_{j|i}^{\ell }=V^{\ell }\left({\tilde{h}}_{j|i}^{\;\ell }\right), \end{array}$$

where $Q^{\ell }$, $K^{\ell }$, and $V^{\ell }$ are learnable feed‐forward projections. The attention coefficient assigned to neighbor j when updating residue i is given by

(9) $$\begin{array}{c} e_{ij}^{\ell }=\frac{Q_{i}^{\ell }{\left(K_{j|i}^{\ell }\right)}^{T}}{\sqrt{d_{\ell }}},\;\alpha_{ij}^{\ell }=\frac{\exp(e_{ij}^{\ell })}{\sum_{k\in N(i)}\exp\left(e_{ik}^{\ell }\right)}, \end{array}$$

where $d_{\ell }$ is the feature dimension of the ℓ‐th attention block and N(i) denotes the neighbor set of residue i. The updated representation of residue i is then obtained as

(10) $$\begin{array}{c} h_{i}^{\ell +1}=\sum_{j\in N(i)}\alpha_{ij}^{\ell }V_{j|i}^{\ell }. \end{array}$$

For multi‐head attention, this procedure is performed independently across t attention heads, and the outputs of all heads are concatenated to form the final updated representation.

#### 4.3.3 Structure‐Wise Message Passing via Geometric Attention

To capture intra‐conformation residue interactions, we perform *structure‐wise message passing* within each conformation. This operation updates residue representations by aggregating information from spatial neighbors under geometric constraints.

For residue i in conformation k, the updated representation is computed as

(11) $$h_{i}^{(k)\prime }=\mathrm{GeoAttn}\;\left(h_{i}^{\left(k\right)},\left\{h_{j}^{\left(k\right)},x_{j}^{\left(k\right)},R_{j}^{\left(k\right)}|j\in N_{i}^{\left(k\right)}\right\}\right),$$

where $N_{i}^{\left(k\right)}$ denotes the spatial neighborhood of residue i in conformation k.

Structure‐wise message passing enables the model to capture local geometric patterns, such as residue packing, secondary structure organization, and conformation‐specific contacts. Importantly, message passing is restricted to residues within the same conformation, ensuring that all geometric relationships remain physically meaningful.

#### 4.3.4 Ensemble‐Wise Message Passing Across Conformations

To explicitly model conformational heterogeneity, we introduce *ensemble‐wise message passing*, which allows information exchange across different conformations of the same residue.

For each residue i, its representations across all conformations

(12) $$\left\{h_{i}^{\left(1\right)},h_{i}^{\left(2\right)},\cdots ,h_{i}^{\left(K\right)}\right\}$$

are treated as nodes in an ensemble‐level graph. Message passing is then performed across conformations, while preserving residue identity.

Specifically, ensemble‐wise attention aggregates information from alternative conformations as

(13) $${\tilde{h}}_{i}^{\left(k\right)}=\mathrm{GeoAttn}\;\left(h_{i}^{\left(k\right)},\left\{h_{i}^{\left(k^{\prime }\right)},x_{i}^{\left(k^{\prime }\right)},R_{i}^{\left(k^{\prime }\right)}|k^{\prime }\in C_{i}\right\}\right),$$

where $C_{i}$ denotes the set of conformations associated with residue i.

Unlike structure‐wise message passing, ensemble‐wise attention operates across conformations at fixed residue identity. This mechanism allows the model to integrate alternative geometric realizations of one residue, enabling it to distinguish stable structural features from conformation‐specific fluctuations and to learn ensemble‐aware residue representations.

### 4.4 Conformational Coverage Metrics

In DyProL, protein dynamics are approximated by a finite ensemble of representative conformations selected from a larger set of sampled equilibrium structures. A key methodological question is whether a compact ensemble sufficiently represents the underlying conformational space, and how the number of representative conformations should be chosen to balance coverage and computational efficiency. To address these questions quantitatively, we introduce two complementary metrics: the *coverage rate* and the *Area under the Coverage Curve* (AUCC). Together, these metrics measure how well a selected conformational subset represents a larger reference ensemble across different structural tolerances. Higher AUCC values indicate that a representative ensemble provides consistently high coverage across a broad range of RMSD tolerances, reflecting both completeness and robustness of conformational representation.

**Coverage rate**. Let $E=\{E_{1},E_{2},\cdots ,E_{M}\}$ denote a reference ensemble of protein conformations sampled by BioEmu, and let $R=\{R_{1},R_{2},\cdots ,R_{K}\}$ denote a smaller representative set of conformations selected from, or constructed from, E. For a given RMSD threshold τ, we define an indicator function for each reference conformation $E_{i}$ as

(14) $$I_{\tau }\left(E_{i},R\right)=\left\{\begin{array}{cc} 1, & \text{if}\;\min_{R_{j}\in R}\mathrm{RMSD}\left(E_{i},R_{j}\right)\leq \tau ,\\ 0, & \text{otherwise}. \end{array}\right.$$

The coverage rate at threshold τ is then defined as

(15) $$\mathrm{Coverage}\left(\tau \right)=\frac{1}{M}\sum_{i=1}^{M}I_{\tau }\left(E_{i},R\right),$$

which measures the fraction of conformations in the reference ensemble that are within an RMSD tolerance τ of at least one representative conformation. Thus, the coverage rate quantifies how well the representative set captures the conformational space spanned by the reference ensemble at a specified structural resolution.

**Area under the coverage curve**. Because the measured coverage depends on the RMSD threshold, we evaluated coverage over a series of thresholds $\left\{\tau_{1},\tau_{2},\cdots ,\tau_{L}\right\}$, yielding a coverage curve

(16) $$C={\left\{(\tau_{\ell },\mathrm{Coverage}\left(\tau_{\ell }\right))\right\}}_{\ell =1}^{L}.$$

To summarize ensemble representativeness across multiple structural tolerances using a single scalar, we define the area under the coverage curve (AUCC) as

(17) $$\mathrm{AUCC}=\int_{\tau_{\min}}^{\tau_{\max}}\mathrm{Coverage}\left(\tau \right)\;d\tau .$$

In practice, AUCC was computed from the discrete coverage curve using the auc function in scikit‐learn.metrics [48], which estimates the area under the curve by trapezoidal integration over the sampled RMSD thresholds.

### 4.5 Statistical Analysis

Statistical significance was assessed using a protein‐level paired sign test. The protein, rather than the residue, was used as the unit of analysis to avoid treating residues from the same protein as independent observations. For each dataset, evaluation metric, and comparison setting, we compared the per‐protein AUPRC or AUROC of DyProL with that of the corresponding baseline or competing method. A protein was counted as a DyProL win when DyProL achieved a higher per‐protein metric than the corresponding baseline or competing method.

Under the null hypothesis that the Dynamic model is not more likely than the Static baseline to perform better on an individual protein, the number of Dynamic wins follows a binomial distribution with success probability 0.5. Because our hypothesis was directional, one‐sided exact sign‐test p‐values were computed. Exact ties, if present, were treated as non‐wins for the Dynamic model. The resulting p‐values were adjusted for multiple comparisons using the Benjamini–Hochberg false discovery rate procedure across all Dynamic‐versus‐Static comparisons. Adjusted values are reported as q‐values, with q<0.05 considered statistically significant.

### 4.6 Implementation Details

**Model training**. The model was trained using the binary cross‐entropy loss. Optimization was performed with Adam optimizer, with an initial learning rate of $1\times 10^{-4}$. The learning rate was reduced by a factor of 0.1 if the validation performance did not improve for 5 consecutive epochs. Early stopping was applied when no improvement was observed for 10 consecutive epochs.

**Software packages**. The framework was implemented using standard scientific computing and machine learning libraries. Protein structures in PDB format were parsed using Biopython [49]. Molecular dynamics trajectories in XTC format were processed and structurally aligned using MDAnalysis [50] to remove global rigid‐body motions. Conformational ensembles were clustered on the basis of structural similarity using scikit‐learn [48] to select representative conformations. All neural network components, including the geometric attention modules and multi‐conformation message‐passing layers, were implemented and trained in PyTorch [51].

**Computing platform**. Model training and evaluation were conducted on a workstation equipped with an Intel 12th Gen Core i9‐12900 CPU, 128 GB RAM, and an NVIDIA RTX A5000 GPU with 24 GB of memory. Large‐scale structure generation and preprocessing, including AlphaFold structure prediction and conformational ensemble generation using BioEmu, AlphaFlow, ESMFlow, and ESMDiff, were performed on a high‐performance server equipped with an Intel Xeon Gold 5320 CPU at 2.20 GHz, 512 GB RAM, and four NVIDIA A800 GPUs with 80 GB of memory each. Detailed profiling of computational cost and memory usage for the full DyProL pipeline is provided in Note S5.

## Author Contributions

Pengpai Li and Yiman Liu conceived the study and designed the research. Pengpai Li and Yiman Liu performed the experiments, analyzed the data, and wrote the manuscript. Liya Liang and Rongming Liu supervised the study, revised the manuscript, and provided overall guidance for the project. All authors contributed to the article and approved the final version of the manuscript.

## Conflicts of Interest

The authors declares no conflicts of interest.

## Supporting information


**Supporting File**: advs77501‐sup‐0001‐SuppMat.pdf

## Data Availability

All code used for model training, evaluation, and comparative analyses has been made publicly available at GitHub https://github.com/Aolibaba/DyProL. The processed data files and protein lists required to reproduce the training and benchmarking experiments are also provided through the same repository. The dynamic structural datasets generated in this study have been deposited in Zenodo https://zenodo.org/records/19547616, including 100 conformations per protein sampled or generated by BioEmu, AlphaFlow, ESMFlow, and ESMDiff. These data are provided as a resource for reproducing DyProL and future development of protein dynamic representation methods by the broader community.
