# Supplementary material for: DyProL: Dynamic Ensemble Representation Learning for Protein–Nucleic Acid Binding Site Prediction
Source: Adv Sci (Weinh). 2026 Sep 3:e77501. Online ahead of print. doi: 10.1002/advs.77501 (PMC13542412; doi:10.1002/advs.77501)
Supplement: Supplementary file 1 [file ADVS-9999-e77501-s001.pdf]

Supplementary information for

DyProL: dynamic ensemble representation learning for protein–nucleic acid binding site prediction

Pengpai Li, Yiman Liu, Liya Liang, Rongming Liu

*Corresponding author(s). E-mail(s): Rongming_Liu@dlut.edu.cn;

This PDF file includes:

Supplementary Notes 1 to 5

Table S1 to S5

Figs. S1 to S12

SI References

Supplementary Information

Note 1. Algorithm 1 description. This algorithm describes the overall multi-conformation learning framework for residue-level binding-site prediction. Given an ensemble of K protein conformations, each conformation is represented by residue features, coordinates, and local reference frames. The model iteratively refines residue representations through B stacked blocks, where each block consists of two stages: intra-conformation aggregation and inter-conformation aggregation. The intra-conformation stage captures local geometric interactions within each individual structure, while the inter-conformation stage enables information exchange across different conformations, allowing the model to learn consistent patterns from structural variability. Both stages are implemented using the geometric attention module (GeoAttentionBlock), ensuring parameter efficiency and unified geometric reasoning. After iterative updates, the final representations from all conformations are aggregated using a conformation-attentive pooling mechanism, which assigns adaptive weights to different conformations based on their relevance. The pooled residue representations are then passed through a classification head to produce binding-site logits and probabilities. This design allows the model to integrate structural heterogeneity and capture both stable and transient binding signals across conformational ensembles.

Algorithm 1: Multi-Conformation Learning Network

Data: Input residue feature: $\mathbf{f}_{in} \in \mathbb{R}^{K\times N\times N_i}$
Input coordinate: $\mathbf{x} \in \mathbb{R}^{K\times N\times 3}$
Input local frame: $\mathbf{l} \in \mathbb{R}^{K\times N\times 3\times 3}$
Intra-conformation neighbor index: $\mathbf{nn}_{intra} \in \mathbb{N}^{K\times N\times M}$
Inter-conformation neighbor index: $\mathbf{nn}_{inter} \in \mathbb{N}^{K\times N\times M_c}$
Number of blocks: B
Result: Prediction logits: $\mathbf{y} \in \mathbb{R}^{N\times 2}$
Prediction probability: $\mathbf{p} \in \mathbb{R}^{N\times 2}$

$\mathbf{h}^{(0)} \leftarrow \mathbf{f}_{in}$; `// initialize hidden features`
for $b \leftarrow 1$ **to** B **do**
  `// Intra-conformation geometric aggregation`
  $\widetilde{\mathbf{h}}^{(b)} \leftarrow \text{GeoAttentionBlock}\big(\mathbf{h}^{(b-1)}, \mathbf{x}, \mathbf{l}, \mathbf{nn}_{intra}\big) \in \mathbb{R}^{K\times N\times N_d}$
  `// Inter-conformation geometric aggregation`
  $\mathbf{h}^{(b)} \leftarrow \text{GeoAttentionBlock}\Big(\widetilde{\mathbf{h}}^{(b)}, \mathbf{x}, \mathbf{l}, \mathbf{nn}_{inter}\Big) \in \mathbb{R}^{K\times N\times N_d}$
end
`// Conformation-attentive pooling`
$\bar{\mathbf{h}} \leftarrow \frac{1}{K}\sum_{k=1}^{K} \mathbf{h}^{(B)}_{k,:,:} \in \mathbb{R}^{N\times N_d}$; `// mean representation over conformations`
$\alpha \leftarrow \text{softmax}(\frac{\langle \mathbf{h}^{(B)}, \bar{\mathbf{h}}\rangle}{\sqrt{N_d}}) \in \mathbb{R}^{K\times N}$; `// weights for residues across conformations`
$\mathbf{z} \leftarrow \sum_{k=1}^{K} \alpha_k \odot \mathbf{h}^{(B)}_{k,:,:} \in \mathbb{R}^{N\times N_d}$; `// pooled residue representation`
`// Residue-level classification`
$\mathbf{y} \leftarrow \text{MLP}_{cls}(\mathbf{z}) \in \mathbb{R}^{N\times 2}$; `// binding-site logits`
$\mathbf{p} \leftarrow \text{Softmax}(\mathbf{y}) \in \mathbb{R}^{N\times 2}$; `// binding-site probabilities`

Note 2. Algorithm 2 description. This algorithm defines a geometry-aware attention mechanism for residue-level interaction modeling. Node and neighbor features are projected into a shared latent space, and relative geometry—including local-frame positions and orientations—is explicitly encoded. A learnable distance-dependent gate modulates neighbor contributions. Multi-head attention is then applied, where queries are derived from central residues and keys/values from neighbors, enabling adaptive aggregation of spatially relevant information. The aggregated features are transformed and combined with residual connections

and normalization to produce updated node representations. This design integrates geometric structure into attention, enabling effective modeling of complex three-dimensional interactions.

Algorithm 2: Geometric Attention Block (GeoAttentionBlock)

Data: Node feature: $\mathbf{f}_{in} \in \mathbb{R}^{N \times N_i}$
Node coordinate: $\mathbf{x} \in \mathbb{R}^{N \times 3}$
Local frame: $\mathbf{l} \in \mathbb{R}^{N \times 3 \times 3}$
Neighbor index: $\mathrm{nn} \in \mathbb{N}^{N \times M}$
Result: Updated node feature: $\mathbf{f}_{out} \in \mathbb{R}^{N \times N_d}$

// Feature projection
$\mathbf{f} \leftarrow W_c(\mathbf{f}_{in}) \in \mathbb{R}^{N \times N_d}$; // project input features to hidden dimension

// Neighbor feature projection
$\mathbf{f}_{nn} \leftarrow \mathbf{f}_{in}[\mathrm{nn}] \in \mathbb{R}^{N \times M \times N_i}$; // gather neighbor features
$\mathbf{f}_{nn} \leftarrow W_n(\mathbf{f}_{nn}) \in \mathbb{R}^{N \times M \times N_d}$; // map to hidden space

// Multi-head query
$\mathbf{Q} \leftarrow f_{query}(\mathbf{f}) \in \mathbb{R}^{N \times N_d}$; // compute query embedding
$\mathbf{Q} \leftarrow \mathrm{reshape}(\mathbf{Q}) \in \mathbb{R}^{N \times N_h \times d_h}$; // split into N_h heads

// Relative geometry encoding
$\mathbf{x}_{nn} \leftarrow \mathbf{x}[\mathrm{nn}] \in \mathbb{R}^{N \times M \times 3}$; // neighbor coordinates
$\Delta\mathbf{x} \leftarrow \mathbf{x}_{nn} - \mathbf{x}[:, \mathrm{None}, :] \in \mathbb{R}^{N \times M \times 3}$; // relative position
$\mathbf{p}_{rel} \leftarrow \mathrm{matmul}\big(\mathbf{l}, \Delta\mathbf{x}^\top\big)^\top \in \mathbb{R}^{N \times M \times 3}$; // coordinates in local frame
$\mathbf{l}_{nn} \leftarrow \mathbf{l}[\mathrm{nn}] \in \mathbb{R}^{N \times M \times 3 \times 3}$; // neighbor frames
$\mathbf{R}_{rel} \leftarrow \mathrm{matmul}\big(\mathbf{l}^\top[:, \mathrm{None}, :, :], \mathbf{l}_{nn}\big) \in \mathbb{R}^{N \times M \times 3 \times 3}$; // relative rotation
$\mathbf{R}_{rel} \leftarrow \mathrm{reshape}(\mathbf{R}_{rel}) \in \mathbb{R}^{N \times M \times 9}$; // flatten rotation matrix
$\mathbf{g} \leftarrow f_{geo}([\mathbf{p}_{rel} \parallel \mathbf{R}_{rel}]) \in \mathbb{R}^{N \times M \times N_d}$; // encode geometric features

// Learnable distance gate
$\mathbf{d} \leftarrow \|\Delta\mathbf{x}\|_2 \in \mathbb{R}^{N \times M \times 1}$; // pairwise distance
$\gamma \leftarrow \sigma(f_{dist}(\mathbf{d})) \in \mathbb{R}^{N \times M \times N_d}$; // distance-dependent gating

// Feature fusion
$\mathbf{f}_{nn} \leftarrow \mathbf{g} \odot \gamma \odot \mathbf{f}_{nn} \in \mathbb{R}^{N \times M \times N_d}$; // geometry-modulated neighbor features

// Key / Value
$\mathbf{K} \leftarrow f_{key}(\mathbf{f}_{nn}) \in \mathbb{R}^{N \times M \times N_d}$; // key projection
$\mathbf{V} \leftarrow f_{value}(\mathbf{f}_{nn}) \in \mathbb{R}^{N \times M \times N_d}$; // value projection
$\mathbf{K} \leftarrow \mathrm{reshape}(\mathbf{K}) \in \mathbb{R}^{N \times N_h \times M \times d_h}$; // multi-head keys
$\mathbf{V} \leftarrow \mathrm{reshape}(\mathbf{V}) \in \mathbb{R}^{N \times N_h \times M \times d_h}$; // multi-head values

// Attention
$\alpha \leftarrow \mathrm{Softmax}\Big(\frac{\langle \mathbf{Q}, \mathbf{K} \rangle}{\sqrt{d_h}}\Big) \in \mathbb{R}^{N \times N_h \times M}$; // attention weights over neighbors
$\mathbf{C} \leftarrow \sum_{j=1}^{M} \alpha_j \odot \mathbf{V}_j \in \mathbb{R}^{N \times N_h \times d_h}$; // aggregate neighbor information
$\mathbf{C} \leftarrow \mathrm{reshape}(\mathbf{C}) \in \mathbb{R}^{N \times N_d}$; // merge attention heads

// Output
$\mathbf{f}_{update} \leftarrow f_{out}(\mathbf{C}) \in \mathbb{R}^{N \times N_d}$; // output transformation
$\mathbf{f}_{out} \leftarrow \mathrm{LayerNorm}(\mathbf{f}_{update} + \mathbf{f}) \in \mathbb{R}^{N \times N_d}$; // residual connection and normalization

Note 3. Benchmarking setup and comparative evaluation

3.1 **Integrating sequence features into DyProL**. DyProL is a general framework that enables flexible integration of heterogeneous information into node-level representations. To ensure that DyProL achieves its optimal predictive performance in comparison experiments, we incorporate both sequence-derived and evolutionary features into the model, as adopted in EquiPNAS[1]. Specifically, we utilize protein language model embeddings generated by ESM2[2] (esm2_t48_15B_UR50D), which encode contextualized sequence representations learned from large-scale protein data. In addition, we incorporate evolutionary information derived from multiple sequence alignments (MSAs). MSAs are generated from the input amino acid sequences using the ColabFold[3] pipeline, which employs MMseq2[4] for efficient sequence retrieval and alignment construction. The resulting MSA is then processed through the Evoformer module of AlphaFold2[5], as implemented in ColabFold, to obtain a distilled MSA representation. We extract the first-row representation ("msa_first_row") as the MSA feature for each protein. For structural information, DyProL directly operates on multi-conformation protein inputs and learns geometric features implicitly through its architecture, without requiring additional handcrafted structural descriptors. These features are integrated into DyProL only for comparison with existing methods, ensuring that the model is evaluated under a setting that maximizes its predictive capability while maintaining fairness in benchmarking.

3.2 **Datasets curated by GraphBind**. The datasets curated by GraphBind have been widely adopted as benchmark datasets for nucleic acid binding-site prediction and have been extensively validated in previous studies. We therefore use these datasets to evaluate the performance of DyProL.

3.3 **Training and evaluation protocol**. We benchmarked DyProL against several representative methods, including EquiPNAS[1], GraphSite[6], GeoNet[7], and GraphBind[8].

For all deep learning–based methods (DyProL, EquiPNAS, GraphSite, GeoNet, and GraphBind), we employed a unified validation strategy to ensure a fair comparison and to avoid potential information leakage. Specifically, 10% of the training data was held out as a validation set for model selection. The best-performing checkpoint was selected based on validation performance and subsequently evaluated on the independent test set.

Following this protocol, we reimplemented and retrained EquiPNAS (https://github.com/Bhattacharya-Lab/EquiPNAS), GeoNet (https://github.com/hjy23/GeoNet), and GraphBind (http://www.csbio.sjtu.edu.cn/bioinf/GraphBind/) using their official codebases with identical data splits and evaluation settings, and reproduced their reported performance accordingly. For GraphSite, performance metrics were directly obtained from the original publication.

Note 4. A detailed description of the data collection, redundancy reduction, dataset splitting, and train–test sequence identity verification.

To construct non-redundant datasets for DNA- and RNA-binding site prediction, we collected protein–nucleic acid complex entries from the Nucleic Acid Knowledgebase (NAKB; accessed on October 8, 2025). Entries annotated as DNA-binding proteins (DBPs) or RNA-binding proteins (RBPs) were first selected. Only experimentally resolved structures with resolution better than 3.5 Å were retained. This filtering step yielded 7,996 DBP-associated PDB entries and 5,071 RBP-associated PDB entries.

Protein chains were then extracted from these protein–nucleic acid complexes. After chain extraction, the initial chain-level datasets contained 20,293 DBP chains and 19,832 RBP chains. Because many PDB entries contain highly similar homologous proteins, multiple chains from the same protein family, or repeated structures of the same protein, we performed sequence-level redundancy reduction before splitting the data into training, validation, and test sets.

Redundancy reduction was performed using CD-HIT. Rather than applying only a single clustering step, we used a progressive clustering strategy with decreasing sequence identity thresholds. Specifically, the initial chain-level datasets were sequentially clustered at sequence identity thresholds of 0.9, 0.8, 0.7, 0.6, 0.5, 0.4, and 0.3. At each step, only the representative sequences retained by CD-HIT were used as the input for the next clustering step. This progressive procedure gradually removed homologous and redundant proteins and produced final protein-level datasets clustered at 30% sequence identity. The resulting non-redundant datasets contained 1,341 DBPs and 1,227 RBPs.

Because DyProL requires structure prediction and conformational ensemble generation for each protein, very long proteins impose substantial computational costs. We therefore removed proteins longer than 1,000 amino acids. After this length filtering step, the final datasets contained 1,278 DBPs and 1,166 RBPs. The final non-redundant DBP and RBP datasets were randomly divided into training, validation, and test sets, with 20% of proteins used as the test set. From the remaining 80%, 10% was used as the validation set, and the rest was used for training.

To further verify the absence of train–test homology leakage, we calculated the maximum sequence identity between each test-set protein and all training-set proteins using BLASTP. The analyses confirmed that all test–train sequence identities were below 40%, supporting the validity of the non-redundant data split. Supplementary Table S2 and S3 give the maximum pairwise sequence identity for RBP and DBP between test-set sequences and their closest train-set matches identified by BLASTP.

For external benchmarking on the GraphBind datasets, we used the GraphBind training and test sets as an independent benchmark setting. The in-house dataset and the GraphBind dataset were not mixed during model training or evaluation. Specifically, models evaluated on the in-house dataset were trained only on the in-house training set, whereas models evaluated on the GraphBind benchmark were trained using the corresponding GraphBind training set and tested on the GraphBind test set. The GraphBind datasets were also constructed with CD-HIT-based redundancy control at a sequence identity threshold of 0.3. In addition, we checked the sequence similarity between the GraphBind benchmark test proteins and the proteins used for DyProL training in that benchmark setting. No train–test protein pair showed sequence identity greater than 40%.

Note 4.1. Additional temporal and homology-controlled benchmark following MPBind-style data construction To further address the concern that high AUROC values may reflect an insufficiently challenging benchmark or residual train–test homology, we performed an additional strict benchmark experiment following the temporal and homology-filtered data construction strategy introduced in MPBind[9]. This analysis was designed as an independent robustness check, rather than as a replacement for the GraphBind-style DNA- and RNA-specific benchmark evaluation used in the main text.

Specifically, protein–nucleic acid complex entries released before January 1, 2022 were used to construct the training set, whereas newly released protein–nucleic acid complexes after January 1, 2022 were collected as an independent temporal test set. To further minimize homology leakage, each test-set protein was compared against all training-set proteins using BLASTP, and any test protein sharing more than 30% sequence identity with a training protein was removed. This 30% sequence identity cutoff is stricter than the commonly used 40% threshold for controlling train–test redundancy in protein function prediction benchmarks.

Under this temporally separated and homology-controlled setting, DyProL achieved an AUROC of 0.917 and an AUPRC of 0.681 for DNA-binding protein prediction, and an AUROC of 0.913 and an AUPRC of 0.688 for RNA-binding protein prediction. These results indicate that DyProL maintains high predictive performance even under a more stringent temporal split and homology-filtered evaluation protocol. Therefore, the high AUROC values observed in the main GraphBind-style benchmark are unlikely to be explained solely by train–test sequence leakage.

It should be noted that MPBind reports a lower AUROC for a combined protein–nucleic acid binding prediction task, in which DNA- and RNA-binding residues are evaluated together within a broader multitask framework. In contrast, the main benchmark evaluation in this study follows the commonly used DNA-specific and RNA-specific protocols adopted by previous protein–nucleic acid binding-site prediction methods. Therefore, absolute AUROC values are not directly comparable across these different task formulations, dataset compositions, and splitting strategies. Nevertheless, the additional temporal and homology-controlled benchmark supports the robustness of DyProL and suggests that its performance is consistent with the current performance range of modern protein–nucleic acid binding-site predictors under established DNA- and RNA-specific evaluation settings.

Note 5. Computational cost and memory usage.

To assess the practical computational cost of DyProL, we profiled the peak GPU memory usage and wall-clock time for the major stages of the pipeline, including BioEmu-based conformational sampling, DyProL training, and DyProL inference. Before DyProL training and inference, BioEmu was used to generate conformational ensembles for each protein. Based on the protein length distribution of our datasets, as shown in Supplementary Fig. S1, BioEmu sampling required an average of 3.8 min per protein on an NVIDIA A800 80 GB GPU. After conformational sampling, 12 representative conformations were selected for downstream multi-conformation learning.

For the DBP/RBP training setting used in this profiling experiment, DyProL training with 12 representative conformations per protein required 19.2 GB peak GPU memory on an NVIDIA RTX A5000 24 GB GPU. The full model training took approximately 45 min, with each epoch requiring about 1.5 min. Training typically stopped after approximately 30 epochs according to the early-stopping criterion. During inference, DyProL required 3.2 GB peak GPU memory and an average wall-clock time of 0.8 s per protein under the same 12-conformation setting.

Table S1 Summary statistics of DNA- and RNA-binding protein datasets curated by this paper.

Type	Dataset	N_{protein}	N_{pos}	N_{neg}	PN ratio
DNA	DNA-1022_Train	1,022	17,650	283,144	0.062
	DNA-256_Test	256	4,470	71,715	0.062
RNA	RNA-932_Train	932	15,729	256,093	0.061
	RNA-234_Test	234	4,235	63,059	0.067

[a] N_{protein}: number of proteins.
[b] N_{pos}: number of positive residues.
[c] N_{neg}: number of negative residues.
[d] PN ratio: ratio of positive to negative residues.

Table S2: Maximum sequence identity between RBP test-set and train-set sequences; test sequences without BLAST results are not shown because no detectable train-set homologs met the BLAST reporting criteria under the specified search parameters.

Test set sequence	Train set sequence	SeqID	Alignment length	qcov	tcov	Bitscore
6ZYM_u	6D2Z_A	0.18125	160	1.019108	0.851064	19.2
8P8B_k	1W2B_K	0.20661	121	0.806667	0.828767	15.4
3QJJ_A	4C9D_A	0.21053	266	1.094650	1.015267	18.1
8X5D_N	6MUR_F	0.21777	349	0.943243	0.925729	55.5
8ZQH_A	8ZQC_A	0.21788	794	0.874449	0.853763	113
6V4X_C	7DVQ_Y	0.22124	113	0.991228	0.807143	22.7
6XLV_A	4LMZ_A	0.22905	179	0.881773	1.017045	45.8
8D8K_H	8P6P_G	0.23179	151	0.974194	1.070922	40
9DOW_A	1ASY_A	0.23542	480	0.948617	0.979592	80.1
5BZ1_A	5YKI_A	0.23821	403	1.086253	1.052219	108
5O60_P	4ANG_A	0.24074	108	0.857143	0.824427	9.6
6ZXG_j	4ANG_A	0.24545	110	0.948276	0.839695	11.5
5GUH_A	8VAJ_A	0.24568	810	1.067194	0.981818	176
5ZWN_b	9ESH_D	0.25253	99	0.831933	1.031250	36.2
9JSB_A	8FFI_B	0.25263	475	0.983437	1.012793	120
5MMI_3	5GAE_b	0.25455	55	0.916667	0.982143	15.8
8SJ7_A	5YKI_A	0.25984	381	0.943069	0.994778	87
7WKP_A	4AL5_A	0.26452	155	0.928144	0.820106	43.9
5MMI_I	6I0Y_5	0.26866	134	0.978102	0.905405	43.1

Continued on next page

Test set sequence	Train set sequence	SeqID	Alignment length	qcov	tcov	Bitscore
4Z0C_A	3CIY_A	0.27368	665	0.937941	1.006051	93.2
9E8Z_C	9NNH_D	0.28154	547	1.024345	0.994545	182
4F02_A	4LMZ_A	0.28758	153	0.874286	0.869318	50.4
1MZP_A	4QVI_A	0.28856	201	0.943662	0.877729	52
8CVM_z	8FN2_d	0.29310	58	0.935484	0.983051	18.1
8CSR_I	8D8L_K	0.29825	114	0.832117	0.802817	37.7
6HTU_A	7ZLQ_A	0.30986	71	0.934211	0.865854	35.8
2ZZN_A	5WT1_A	0.31073	354	1.053571	1.066265	115
1FJG_F	7BOD_F	0.31250	96	0.950495	0.905660	52
6NEQ_N	8D8K_N	0.31373	102	1.009901	0.902655	49.7
5W0O_A	6YWO_A	0.31922	307	0.908284	0.892442	128
1W2B_O	7NAD_R	0.32308	130	0.902778	0.970149	52
4WC2_A	5HC9_A	0.35038	391	1.065395	0.920000	183
9J89_F	2GXB_B	0.35088	57	0.890625	0.904762	31.6

Table S3: Maximum pairwise sequence identity between DBP test-set sequences and their closest train-set matches identified by BLAST. Test sequences without BLAST results are not included, indicating that no detectable train-set homologs met the BLAST reporting criteria under the specified search parameters.

Test set sequence	Train set sequence	SeqID	Alignment length	qcov	tcov	Bitscore
6ERH_A	9N82_b	0.20256	469	0.957143	0.919608	26.2
4HF1_A	2NP2_A	0.20561	107	0.829457	1.049020	13.1
6EN8_A	7AMN_A	0.20645	155	0.842391	0.820106	32.3
6M75_A	9GPJ_A	0.20946	148	0.886228	0.804348	48.9
8D3L_E	7MI4_E	0.21250	80	0.816327	0.842105	23.5
5K7Z_A	8SUK_D	0.21469	177	0.956757	0.889447	43.5
7CE1_K	1EFA_B	0.21538	325	0.975976	0.984848	38.1
7EL3_A	6C2S_A	0.21970	132	0.956522	0.929577	34.3
8X6G_H	8HKC_E	0.22174	230	0.942623	0.877863	43.9
1U78_A	1XSD_A	0.22549	102	0.990291	0.816000	16.9
9CA0_A	2O59_A	0.22565	616	1.008183	0.995153	104

Continued on next page

Test set sequence	Train set sequence	SeqID	Alignment length	qcov	tcov	Bitscore
8A8J_A	5DNY_B	0.22715	361	1.058651	1.005571	47.4
7R8G_A	8XIH_B	0.22745	765	0.994798	1.040816	153
5VC8_A	7LH9_B	0.23301	103	0.837398	0.880342	28.9
2OAA_B	2Q10_A	0.23394	218	0.879032	0.915966	30.4
9CB5_D	1CBV_L	0.23902	205	0.876068	0.936073	57.4
3A5U_A	3ULP_C	0.23958	96	0.813559	0.827586	35.4
9P1D_E	8FYB_C	0.24026	308	0.968553	0.996764	53.5
6GDR_A	7OBN_A	0.24460	278	1.085938	0.929766	50.1
8SH0_A	7CUH_A	0.24585	301	1.023810	0.986885	48.9
7QEN_A	7QEN_B	0.25751	466	1.002151	1.156328	133
6GML_Z	8RAM_Z	0.25773	485	1.031915	1.130536	136
4I6Z_A	7JU3_B	0.25806	186	0.968750	0.916256	51.6
7C7L_A	8J12_A	0.26000	450	0.894632	1.061321	117
4NDH_A	3SPD_A	0.26131	199	1.105556	0.995000	67.8
3K5M_A	4FM2_A	0.26287	738	0.960938	0.980080	170
5GPC_A	7JU3_B	0.26590	173	0.910526	0.852217	47.4
8XRI_B	5GWK_A	0.27227	797	1.032383	1.100829	245
2O8B_B	8R7E_A	0.27332	761	0.818280	0.922424	212
3D2W_A	7Z0U_A	0.27632	76	1.055556	0.974359	17.7
8IAZ_A	8H1J_A	0.27937	383	0.997396	1.058011	134
9E8Z_A	9NNB_C	0.27985	536	1.017078	0.974545	182
7BZG_A	4HQE_B	0.28283	99	0.900000	0.916667	50.8
1R2Y_A	7Z5A_A	0.28315	279	1.021978	1.056818	53.5
1F0V_A	1M07_A	0.29060	117	0.943548	1.063636	35.8
4MTD_D	7VO0_G	0.29134	127	0.841060	0.969466	43.1
6DVC_F	6JCX_F	0.29268	164	0.942529	0.881720	51.6
4PU3_D	3CRO_L	0.29310	58	0.816901	0.878788	25.4
1OMH_A	3UBT_Y	0.29464	336	1.027523	1.024390	145
7PZB_A	3MZH_A	0.29648	199	0.876652	0.884444	87.4
5D39_A	1BF5_A	0.29960	494	1.012295	0.908088	154
1KX5_D	8UA7_D	0.30000	100	0.819672	0.990099	32.7
4ECW_A	4IR1_F	0.30056	356	0.824074	1.040936	120
9C5G_B	7T8K_A	0.31600	250	0.862069	0.874126	126

Continued on next page

Test set sequence	Train set sequence	SeqID	Alignment length	qcov	tcov	Bitscore
9URH_A	7CSZ_A	0.31613	155	0.875706	0.885714	65.1
8X6D_B	2QHB_A	0.31707	82	0.987952	0.953488	28.5
8ZP7_H	5U0A_E	0.32370	346	0.922667	0.942779	150
1MDY_A	2YPB_B	0.35000	60	0.882353	0.810811	31.6
7MI4_B	8D3L_A	0.36471	340	1.000000	0.991254	199
5VPE_D	1GD2_E	0.36667	60	0.895522	0.923077	26.2
3VKE_A	1ZZI_A	0.38028	71	0.934211	0.865854	45.4

Table S4 Statistical comparison of structural diversity and physical plausibility among four ensemble-generation methods for the 15 DBPs analyzed in Fig. 4b. Values are summarized as median [interquartile range] across the 15 proteins. Overall differences among the four methods were assessed using the Friedman test, with Kendall's W reported as the effect size.

Metric	BioEmu median [IQR]	AlphaFlow median [IQR]	ESMFlow median [IQR]	ESMDiff median [IQR]	Friedman χ^2	Global p value	Kendall's W
Mean pairwise Cα RMSD	5.365 [4.072, 7.356]	3.949 [2.364, 5.491]	4.373 [2.860, 5.336]	8.308 [1.756, 12.146]	15.080	0.00175	0.335
Physical plausibility rate	1.000 [1.000, 1.000]	0.880 [0.798, 0.920]	0.863 [0.720, 0.905]	0.975 [0.740, 0.997]	30.265	1.21×10^{-6}	0.673

Table S5 Post-hoc paired Wilcoxon signed-rank tests for structural diversity and physical plausibility among ensemble-generation methods. Median paired difference was calculated as Method 1 minus Method 2 across the same 15 proteins. Holm-adjusted p values were corrected across the six pairwise comparisons within each metric.

Metric	Method 1	Method 2	Median paired difference	Wilcoxon W	Raw p value	Holm-adjusted p value
Mean pairwise Cα RMSD	BioEmu	AlphaFlow	1.667	4.0	0.000427	0.00256
Mean pairwise Cα RMSD	BioEmu	ESMFlow	1.525	15.0	0.00836	0.0418
Mean pairwise Cα RMSD	BioEmu	ESMDiff	-1.105	36.0	0.188	0.375
Mean pairwise Cα RMSD	AlphaFlow	ESMFlow	-0.034	55.0	0.804	0.804
Mean pairwise Cα RMSD	AlphaFlow	ESMDiff	-4.930	20.0	0.0215	0.0862
Mean pairwise Cα RMSD	ESMFlow	ESMDiff	-4.993	20.0	0.0215	0.0862
Physical plausibility rate	BioEmu	AlphaFlow	0.120	0.0	0.0000610	0.000366
Physical plausibility rate	BioEmu	ESMFlow	0.131	0.0	0.000653	0.00327
Physical plausibility rate	BioEmu	ESMDiff	0.025	0.0	0.00222	0.00887
Physical plausibility rate	AlphaFlow	ESMFlow	0.027	26.5	0.0570	0.171
Physical plausibility rate	AlphaFlow	ESMDiff	-0.045	49.0	0.561	0.561
Physical plausibility rate	ESMFlow	ESMDiff	-0.084	37.0	0.208	0.416

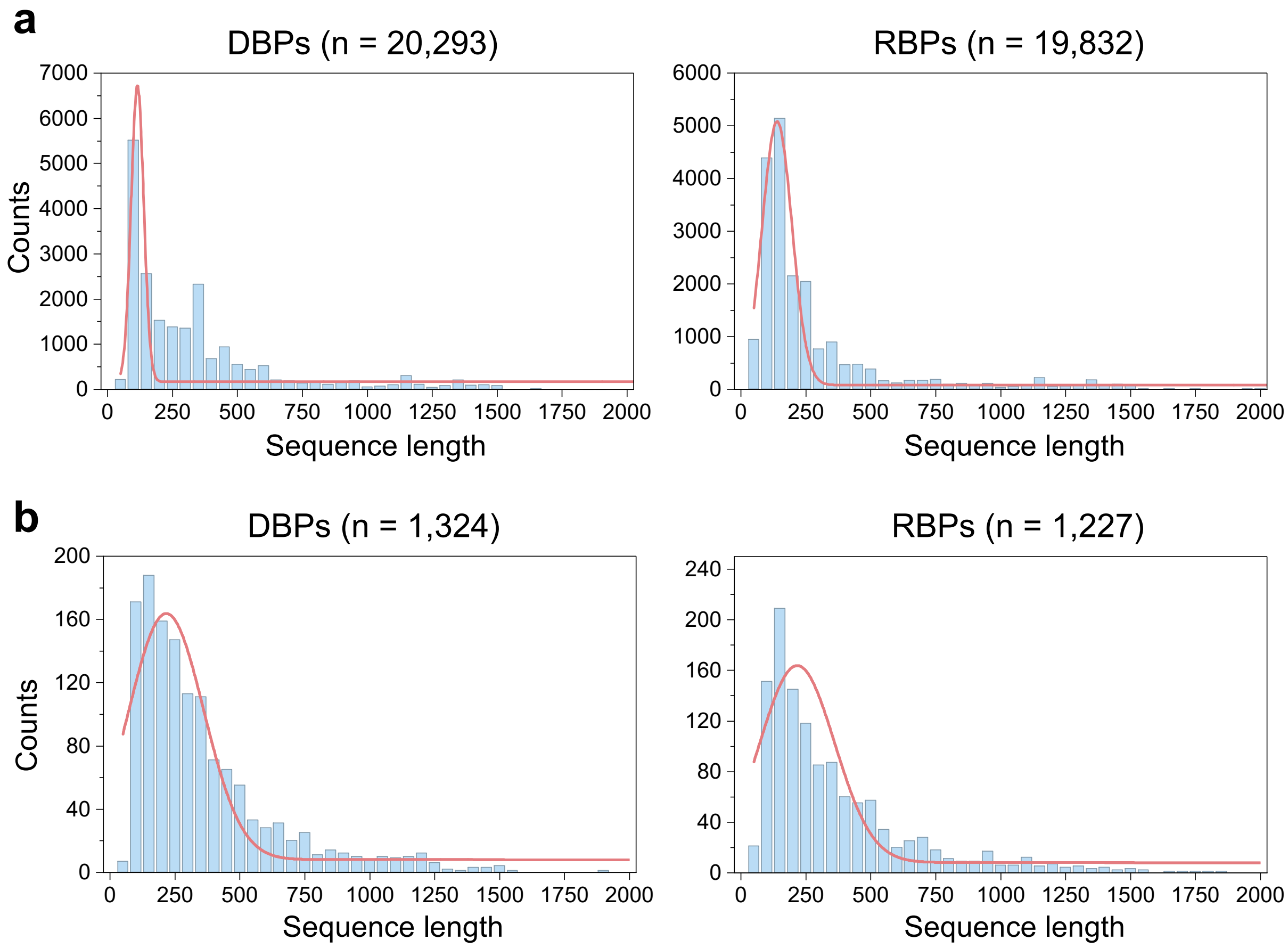

Fig. S1 Sequence length distributions of DNA- and RNA-binding proteins. (a) Sequence length distributions before redundancy reduction for DNA-binding proteins (DBPs) and RNA-binding proteins (RBPs). (b) Sequence length distributions after CD-HIT clustering to remove sequence redundancy (sequence identity threshold = 30%). Red curves indicate kernel density estimates. The overall distribution is largely preserved after redundancy reduction, indicating that clustering does not introduce significant bias in protein length.

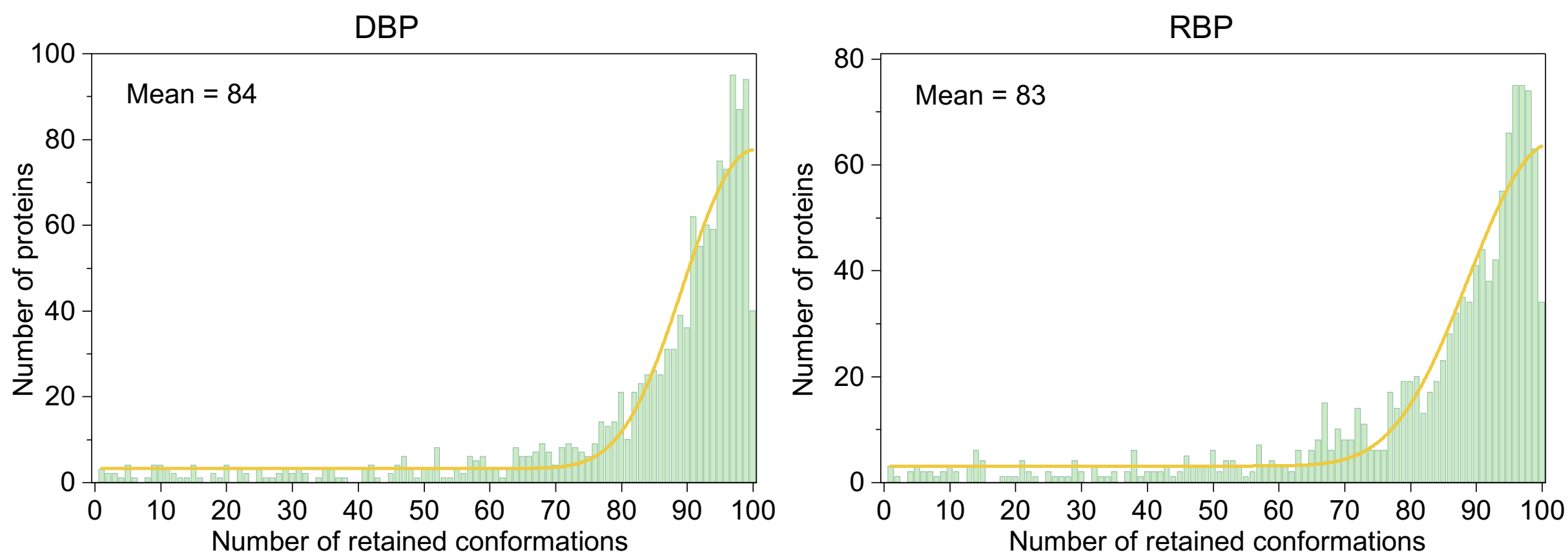

Fig. S2 Distribution of retained conformations after filtering. For each protein, 100 conformations were initially sampled using BioEmu and subsequently filtered to remove physically implausible structures. Histograms show the number of retained conformations per protein for DNA-binding proteins (left) and RNA-binding proteins (right). The mean number of retained conformations is 84 and 83, respectively. The solid curves indicate kernel density estimates. The distributions are strongly skewed toward high values, indicating that most sampled conformations satisfy basic physical constraints.

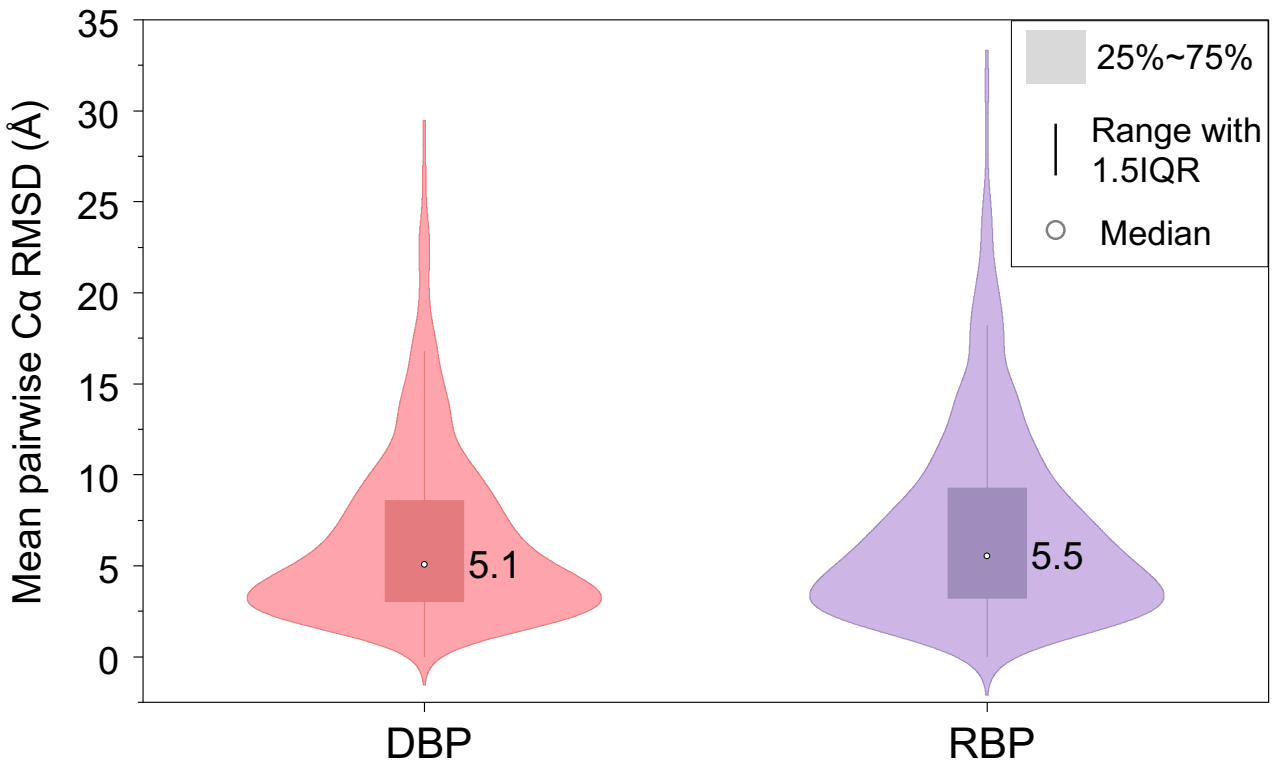

Fig. S3 Mean pairwise Cα RMSD within protein conformational ensembles. For each protein, the mean pairwise Cα RMSD across all sampled conformations is shown for DBPs and RBPs. The average RMSD values are 5.1 Å and 5.5 Å, respectively. These results indicate substantial structural variability within the ensembles, consistent with moderate yet functionally relevant conformational deviations rather than trivial rigid-body fluctuations.

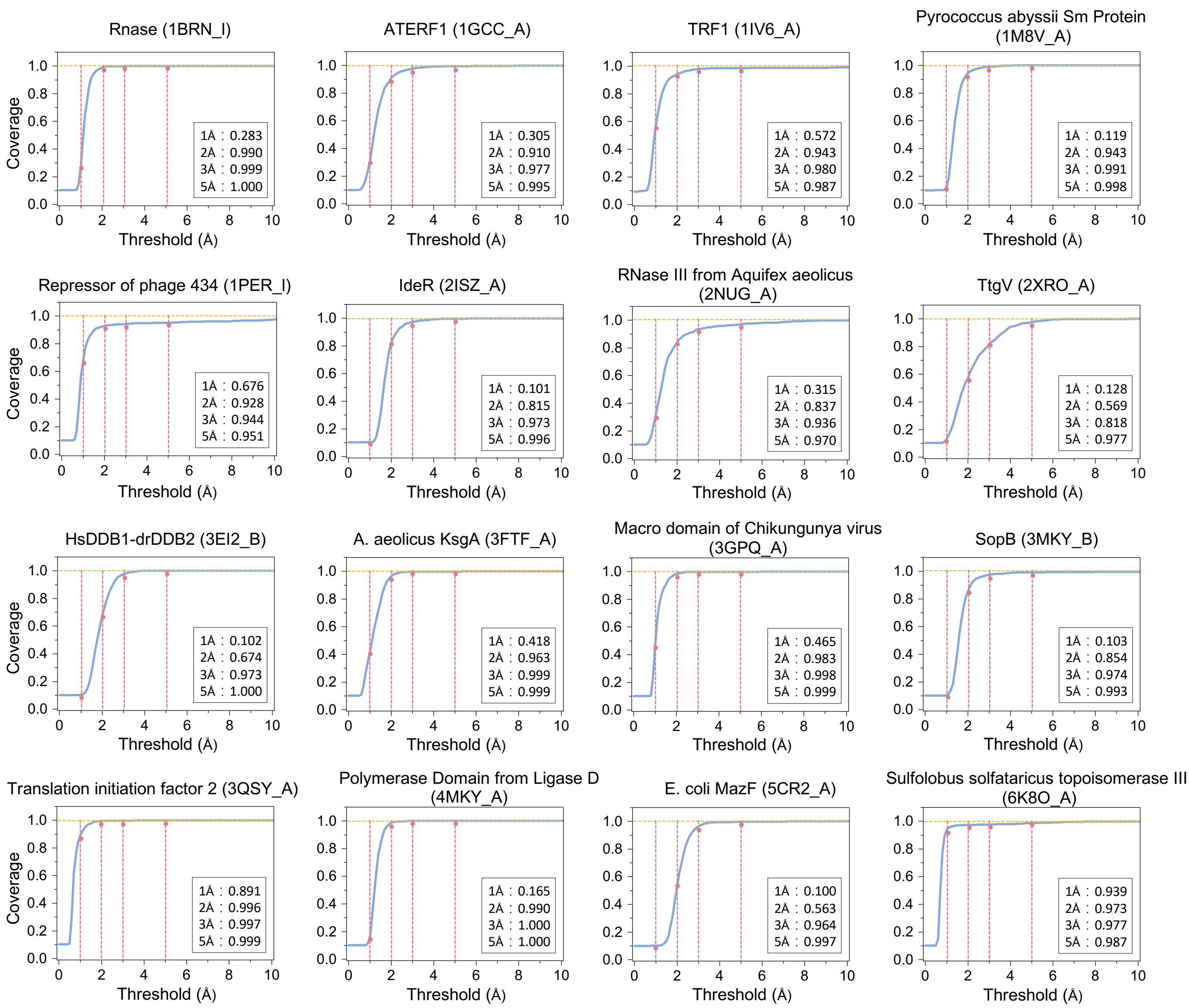

Fig. S4 Coverage of 100-conformation ensembles relative to 1,000-conformation ensembles. Coverage is defined as the fraction of conformations in the 1,000-sample ensemble whose minimum Cα RMSD to any structure in the 100-conformation ensemble is below a given threshold. Results are shown for 16 randomly selected proteins from the DNA-bound-unbound dataset. The coverage increases monotonically with the RMSD threshold and approaches saturation at higher thresholds, indicating that 100 conformations provide a high-fidelity approximation of the conformational space sampled by larger ensembles.

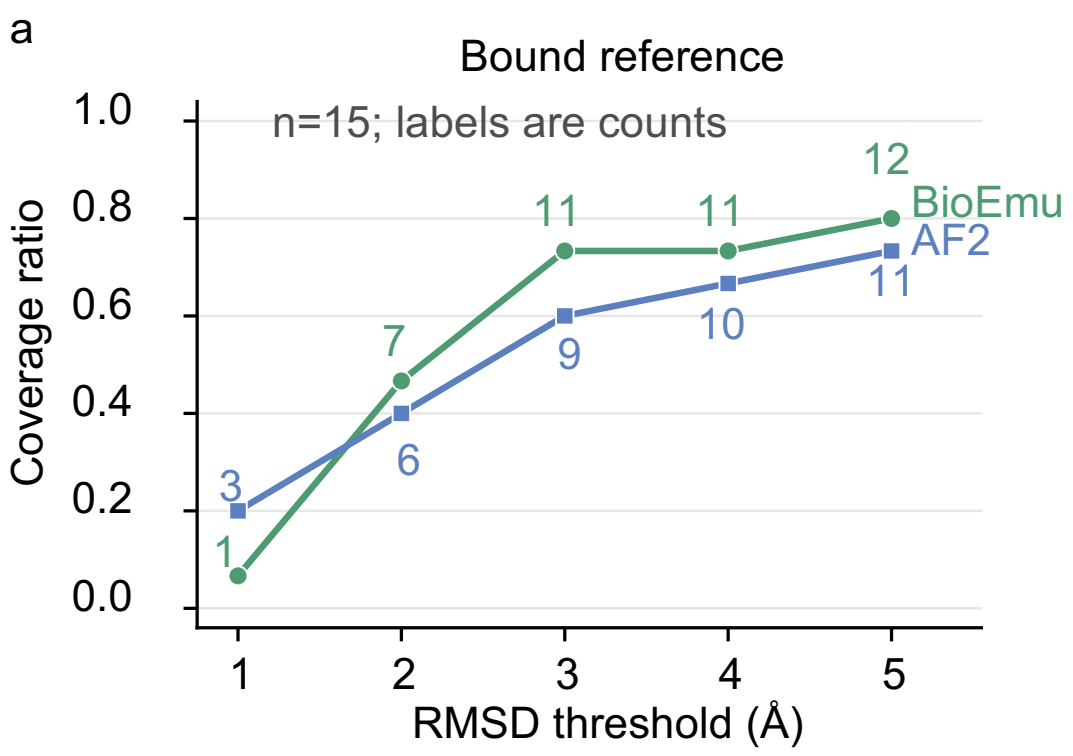

Fig. S5 BioEmu ensembles provide broader protein-level structural coverage than single AlphaFold2 structures. Protein-level coverage was calculated for 15 paired DNA-binding proteins with experimentally resolved bound and unbound reference structures. For each RMSD threshold from 1 to 5 Å, a protein was considered covered by BioEmu if at least one BioEmu conformation had $C\alpha$ RMSD below the threshold after optimal superposition to the reference structure. AlphaFold2 coverage was calculated analogously using the single AlphaFold2-predicted structure. Coverage is shown separately relative to the bound and unbound reference structures. Numbers beside each point indicate the number of covered proteins out of 15. BioEmu achieved higher coverage than AlphaFold2 at most thresholds, particularly relative to the unbound reference, indicating that BioEmu ensembles sample conformations closer to experimentally resolved reference states than a single static AlphaFold2 structure.

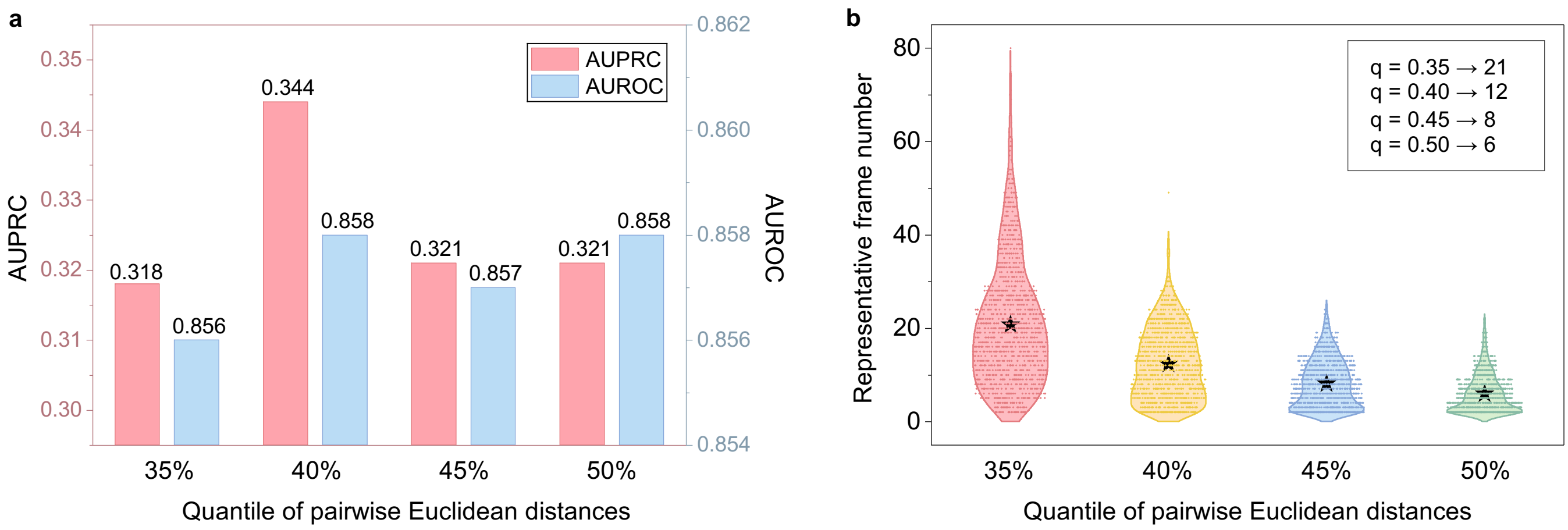

Fig. S6 Performance and stability of DBSCAN-based representative selection. (a) Model performance (AUPRC and AUROC) on the DNA dataset using DBSCAN with different quantiles of the pairwise Euclidean distances between conformation feature vectors (`min_samples`=1). For each protein ensemble, the selected quantile determines the DBSCAN neighborhood radius. Performance varies across quantile settings. (b) Distribution of the number of representative conformations selected per protein under different quantile settings. The number of representatives decreases as the quantile increases and varies substantially across proteins, indicating limited control over cluster granularity and reduced stability of DBSCAN-based selection.

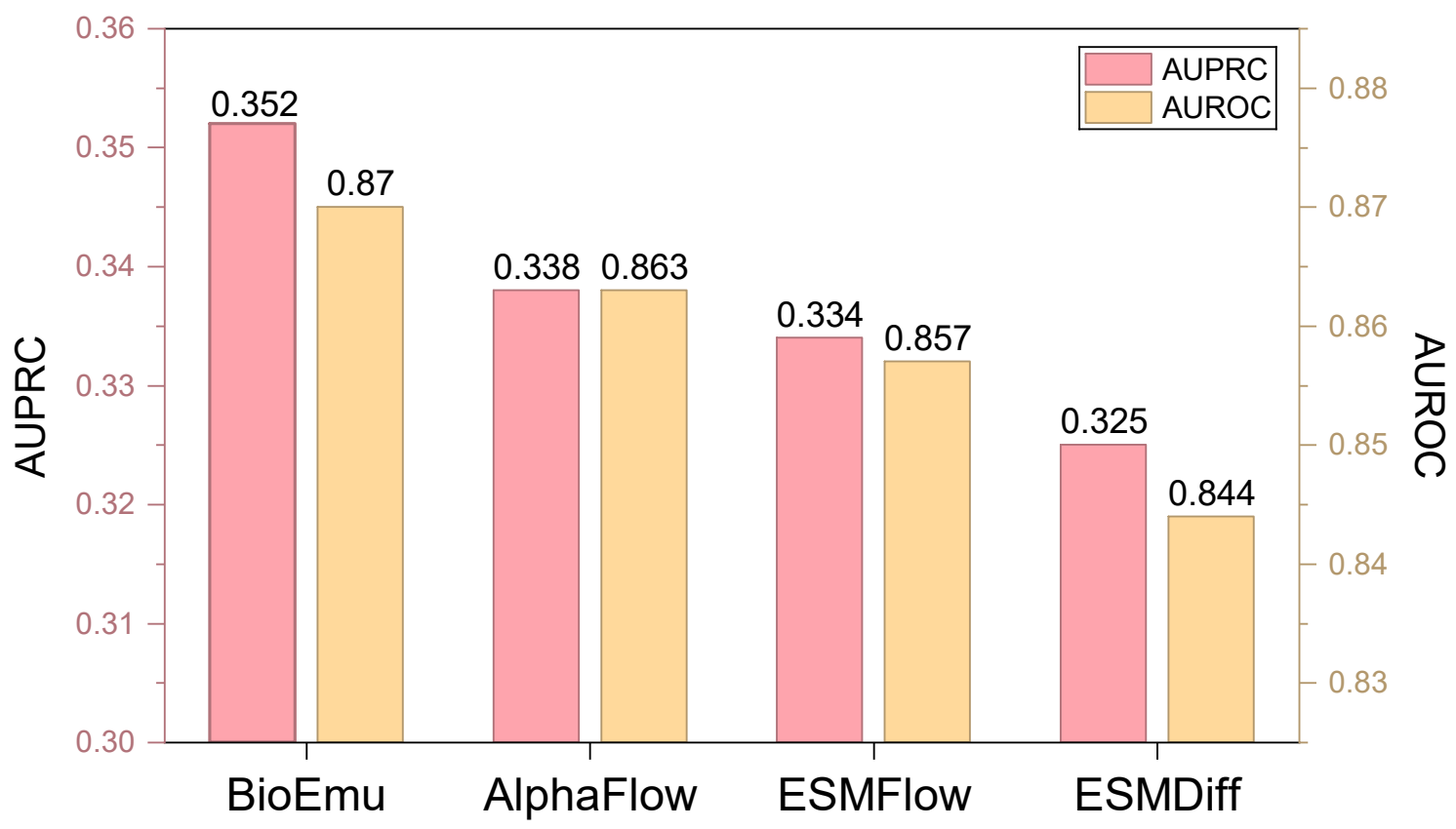

Fig. S7 Overall performance of models trained with different conformational ensembles. Total AUPRC and AUROC on the DBP dataset when training with ensembles generated by BioEmu, AlphaFlow, ESMFlow, and ESMDiff. In contrast to Fig. 4a, which reports per-protein median AUPRC, this figure summarizes performance over all residues across the dataset. The results are consistent with Fig. 4a, with BioEmu achieving the best overall performance, followed by AlphaFlow and ESMFlow, while ESMDiff performs worst.

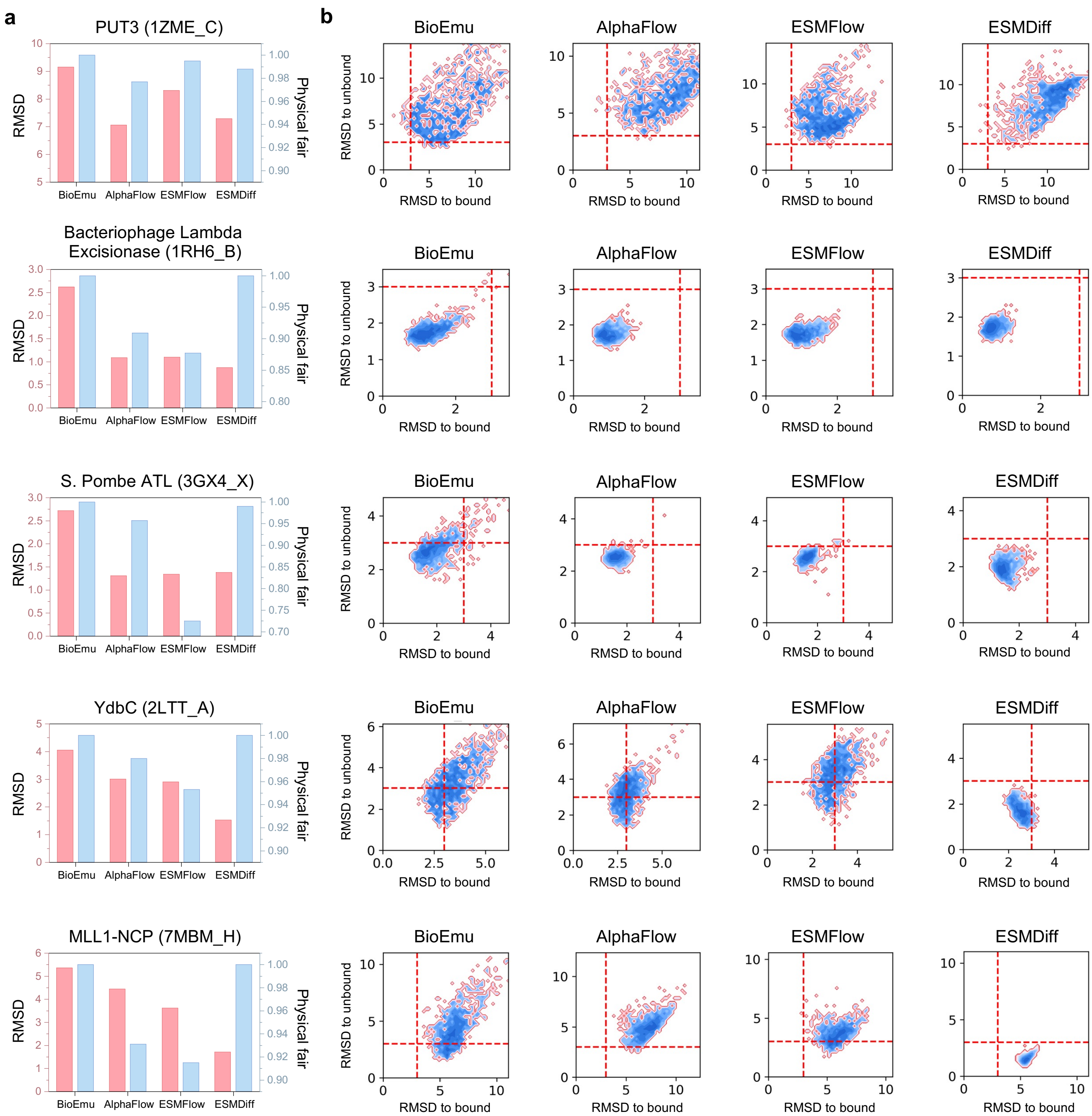

Fig. S8 Conformation ensembles generated by different models for five proteins and their average RMSD and physical plausibility. (a) For the five proteins 1ZME_C, 1RH6_B, 3GX4_X, 2LLT_A and 7MBM_H four models were used to generate different conformation ensembles, and the mean RMSD and physical plausibility of each conformation set for each protein were calculated.(b) 2D free energy maps of different conformational ensembles generated using four models for five proteins.

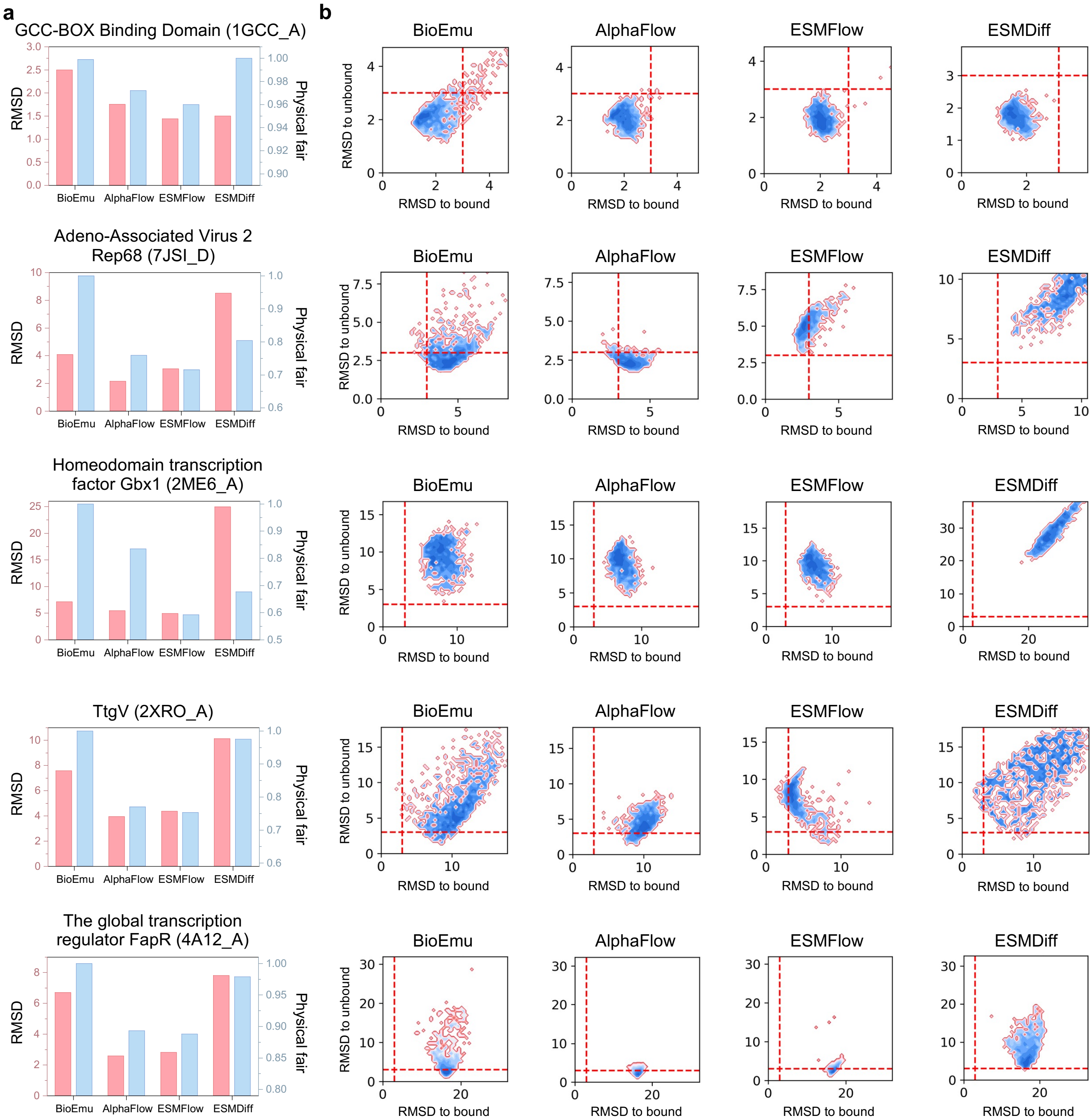

Fig. S9 Conformation ensembles generated by different models for five proteins and their average RMSD and physical plausibility. (a) For the five proteins 1GCC_A, 7JSI_D, 2ME6_A, 2XRO_A and 4A12_A four models were used to generate different conformation ensembles, and the mean RMSD and physical plausibility of each conformation set for each protein were calculated.(b) 2D free energy maps of different conformational ensembles generated using four models for five proteins.

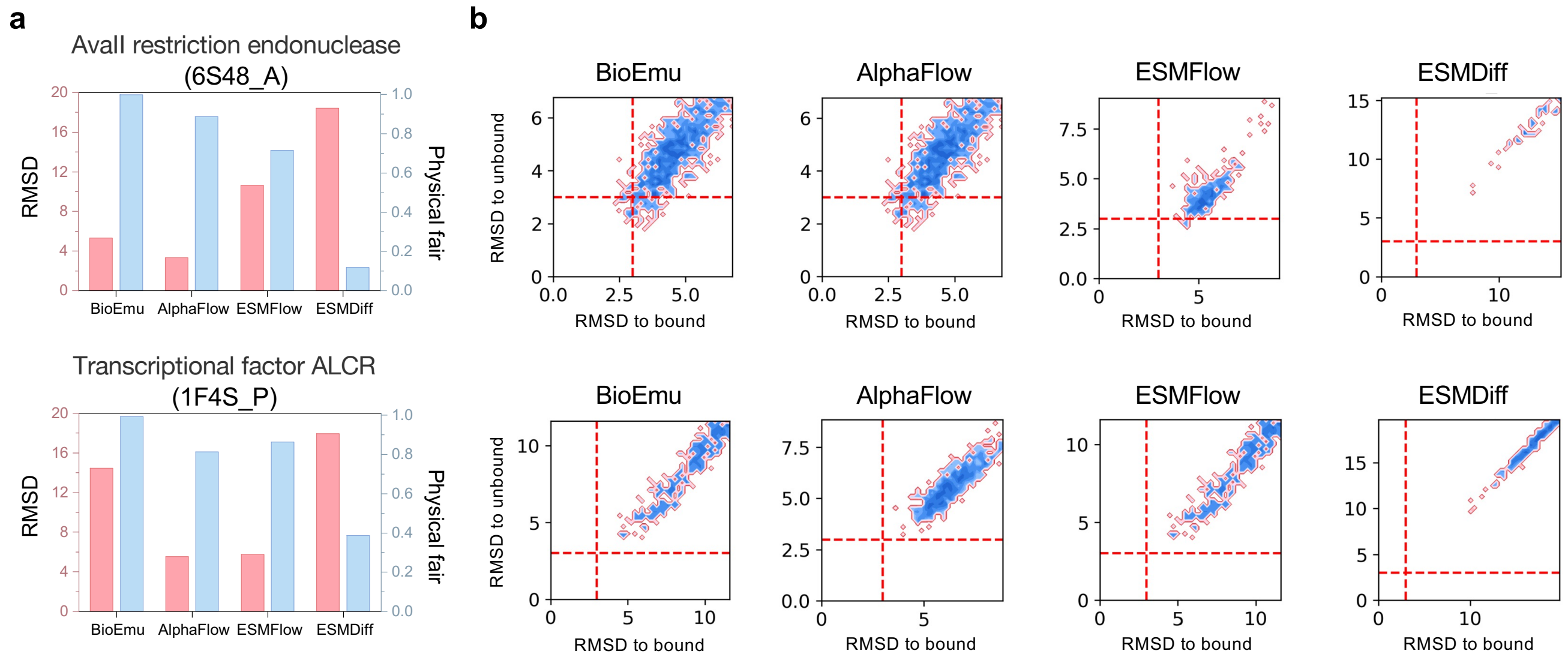

Fig. S10 Conformation ensembles generated by different models for two proteins and their average RMSD and physical plausibility. (a) For the two proteins 6S48_A and 1F4S_P four models were used to generate different conformation ensembles, and the mean RMSD and physical plausibility of each conformation set for each protein were calculated.(b) 2D free energy maps of different conformational ensembles generated using four models for two proteins.

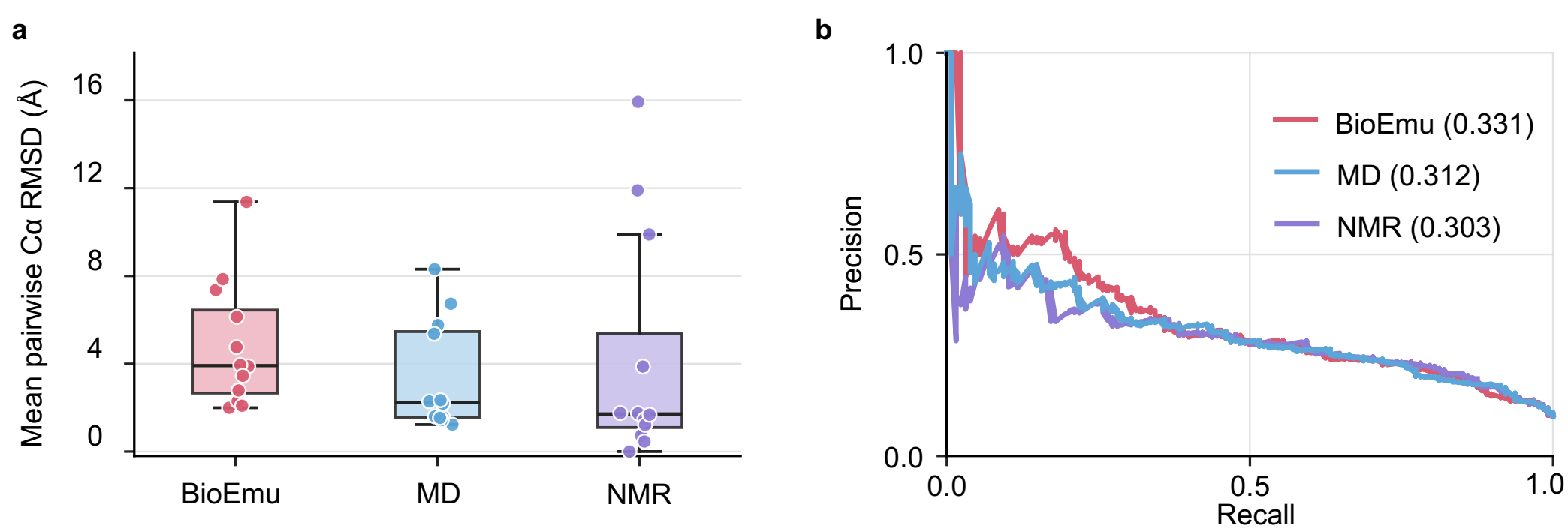

Fig. S11 Comparison of BioEmu-, MD-, and NMR-derived conformational ensembles on the NMR-derived DBP test set. Left, distributions of mean pairwise $C\alpha$ RMSD; right, precision-recall curves and AUPRC values for MD, NMR, and BioEmu conformations.

a

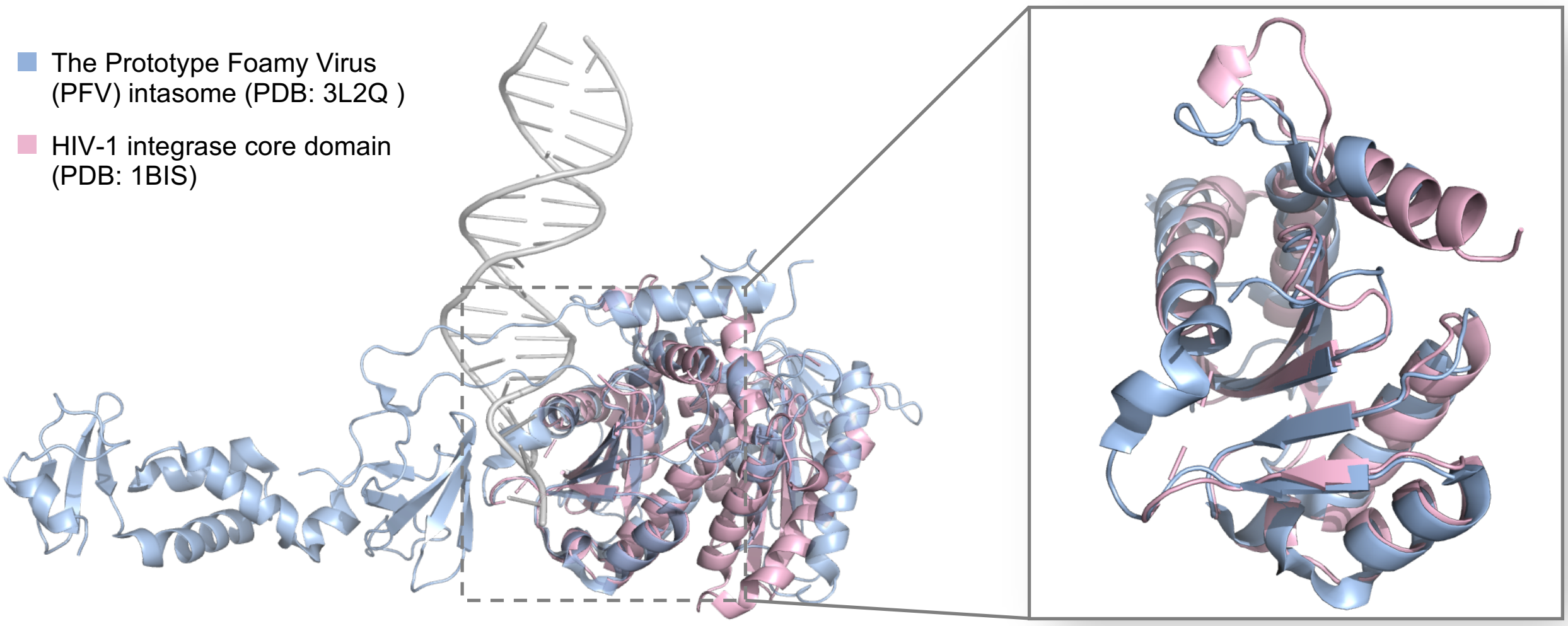

b

```
                 *        20         *        40         *        60         *        80
3L2Q : GPGCNTKKPNLDAELDQLLQGHYIKGYPKQYTYFLEDGKVKVSRPEGVKIIPPQSDRQKIVLQAHNLAHTGREATLLKIAN :  81
1BIS : --------------------GSHMHG--------------QVDCSPGIWQLDCTHLEGKVILVAVHVASGYIEAEVI---- :  43
                           G  6 G               V    G6  6       K66L A  6A    EA 66

               *      100         *      120         *      140         *      160
3L2Q : LYWWPNMRKDVVKQLGRCQQCLITNASNKASGPILRPDRPQKPFDKFFIDYIGPLPPSQGYLYVLVVVDGMTGFTWLYPTK : 162
1BIS : -----------------------------------------------------PAETGQETAYFLLKLAGR------WPVK :  65
                                                            P    Q   Y L6 6 G       5P K

              *      180         *      200         *      220         *      240
3L2Q : APSTSATVKSLNVLTSIAIPKVIHSDQGAAFTSSTF---AEWAKERGIHLEFSTPYHPQSSGKVERKNSDIKRLLTKLLVG : 240
1BIS : T---------------------VHTDNGSNFTSTTVKAACEWA---GIKQEFGIPYNPQSQGVIESMNKELKKIIGQV--- : 119
                             6H3D G  FTS3T     EWA   GI  EF  PY PQS G 6E  N  6K466  6

             *      260         *      280         *      300         *      320
3L2Q : RPTKWYDLLPVVQLALNNTYSPVLKYTPHQLLFGIDSNTPFANQDTLDLTREEELSLLQEIRTSLYHPSTPPASSRSWSPV : 321
1BIS : --------------------------------------------------RDQAEHLKTAVQMAVFIHNKKRKGG-----I : 145
                                                         R 2   L   6   65              6

            *      340         *      360         *      380         *
3L2Q : VGQLVQERVARPASLRPRWHKPSTVLKVLNPRTVVILDHLGNNRTVSIDNLKPTSHQNGTTNDTATMDHLEKNE : 395
1BIS : GGYSAGER---------------------------IVDIIATD----IQDIQ---------------------- : 166
        G    ER                           I6D 6  1    I 16
```

Fig. S12 Structural and sequence comparison between HIV-1 integrase (1BIS_A) and PFV integrase (3L2Q_A). **a**, Structural superposition of the HIV-1 integrase core domain (PDB: 1BIS_A, pink) and the prototype foamy virus (PFV) intasome (PDB: 3L2Q_A, blue). Despite substantial sequence divergence, the two proteins exhibit a highly similar overall fold. The DNA substrate bound to the PFV intasome is shown in gray. The inset highlights the core catalytic region, illustrating the structural conservation of functionally relevant motifs between the two proteins. **b**, Structure-guided sequence alignment between 1BIS_A and 3L2Q_A. Conserved and similar residues are highlighted, and key catalytic residues are indicated. Despite low sequence identity, the alignment reveals conservation at structurally and functionally important positions, supporting the use of PFV integrase as a structural reference for HIV-1 integrase.

References

[1] Roche, R., Moussad, B., Shuvo, M.H., Tarafder, S., Bhattacharya, D.: EquiPNAS: improved protein–nucleic acid binding site prediction using protein-language-model-informed equivariant deep graph neural networks. Nucleic Acids Research **52**(5), 27–27 (2024)

[2] Rives, A., Meier, J., Sercu, T., Goyal, S., Lin, Z., Liu, J., Guo, D., Ott, M., Zitnick, C.L., Ma, J., *et al.*: Biological structure and function emerge from scaling unsupervised learning to 250 million protein sequences. Proceedings of the national academy of sciences **118**(15), 2016239118 (2021)

[3] Mirdita, M., Schütze, K., Moriwaki, Y., Heo, L., Ovchinnikov, S., Steinegger, M.: ColabFold: making protein folding accessible to all. Nature methods **19**(6), 679–682 (2022)

[4] Steinegger, M., Söding, J.: MMseqs2 enables sensitive protein sequence searching for the analysis of massive data sets. Nature biotechnology **35**(11), 1026–1028 (2017)

[5] Jumper, J., Evans, R., Pritzel, A., Green, T., Figurnov, M., Ronneberger, O., Tunyasuvunakool, K., Bates, R., Žídek, A., Potapenko, A., *et al.*: Highly accurate protein structure prediction with AlphaFold. Nature **596**(7873), 583–589 (2021)

[6] Yuan, Q., Chen, S., Rao, J., Zheng, S., Zhao, H., Yang, Y.: AlphaFold2-aware protein–DNA binding site prediction using graph transformer. Briefings in bioinformatics **23**(2), 564 (2022)

[7] Han, J., Zhang, S., Guan, M., Li, Q., Gao, X., Liu, J.: GeoNet enables the accurate prediction of protein-ligand binding sites through interpretable geometric deep learning. Structure **32**(12), 2435–2448 (2024)

[8] Xia, Y., Xia, C.-Q., Pan, X., Shen, H.-B.: GraphBind: protein structural context embedded rules learned by hierarchical graph neural networks for recognizing nucleic-acid-binding residues. Nucleic Acids Research **49**(9), 51–51 (2021)

[9] Wang, Y., Boadu, F., Cheng, J.: MPBind: a multitask protein binding site predictor using protein language models and equivariant GNNs. Bioinformatics **41**(11), 589 (2025)
